# Impact of Structural Heterogeneities in Hierarchically Grown Microribbons on 2D WS_2_ Monolayer on the Measured Local Hydrogen Evolution Activity on Au Substrate

**DOI:** 10.1002/smll.202510517

**Published:** 2026-01-08

**Authors:** Laud Anim Adofo, Alejandro E. Pérez Mendoza, Miran Joo, Lithin Madayan‐Banatheth, Andrew Ben‐Smith, Ki Kang Kim, Christina Scheu, Corina Andronescu

**Affiliations:** ^1^ Chemical Technology III University of Duisburg‐Essen Carl‐Benz‐Straße 199 Duisburg Germany; ^2^ Nanoanalytics and Interfaces Max Planck Institute for Sustainable Materials Düsseldorf Germany; ^3^ Department of Energy Science Sungkyunkwan University Suwon Republic of Korea; ^4^ Center for Nanointegration University of Duisburg‐Essen (CENIDE) Carl‐Benz‐Straße 199 Duisburg Germany

**Keywords:** 2D material, hydrogen evolution reaction, microribbons, scanning electrochemical cell microscopy, transition metal dichalcogenides

## Abstract

Homoepitaxial growth of highly crystalline microribbons on large‐area 2D transition metal dichalcogenide (TMD) monolayers is often facilitated by molten salt chemical vapor deposition (CVD) via the vapor–liquid–solid growth method. These microribbons are vertically stacked TMD monolayer stripes that exhibit well‐defined edge structures, introducing spatial heterogeneities across the basal plane of their 2D monolayer supports. Although frequently observed in CVD‐grown TMDs, their influence on local electrocatalytic behavior remains poorly understood. Here, we use scanning electrochemical cell microscopy (SECCM), combined with correlative microscopy and spectroscopy, to spatially resolve the hydrogen evolution reaction (HER) activity of WS_2_ monolayer decorated with microribbons at nanometer resolution. Quantitative analysis of over 33 000 localized electrochemical measurements reveals that monolayer edges exhibit twice the catalytic activity of the basal plane, while single‐ and multilayer microribbons suppress HER activity by over 5‐ and 10‐fold, respectively, due to interlayer coupling and sluggish charge transport from the underlying Au substrate. Overall, this work reveals how microribbons modulate basal plane activity in 2D WS_2_ on metallic Au substrate and demonstrates how co‐localization of SECCM with various spectroscopies can be used as a powerful technique for mapping nanoscale catalytic activity across structurally complex TMD surfaces.

## Graphical abstract

Spatially resolved SECCM measurements reveal that hierarchically grown microribbons on 2D WS_2_ drastically suppress local HER activity. Relative to the basal plane of the 2D monolayer directly supported on an Au substrate, single‐layer and multilayer microribbons exhibit more than a ∼5‐fold and >10‐fold decrease in current density, respectively. These results highlight thickness‐driven catalytic heterogeneity, with SECCM coupled with various microscopies and spectroscopies enabling precise probing of local reactivity in various structurally complex 2D materials.

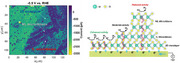

## 1 Introduction

Dimensional engineering of transition metal dichalcogenides (TMDs) represents a compelling strategy to uncover novel quantum phenomena and electronic properties at the atomic scale [1]. For example, 1D nanostructures derived from TMD monolayers, such as MoS_2_ nanoribbons, introduce geometric anisotropy and exhibit a rich array of emergent behaviors, including confined electronic states, ferromagnetic ordering, highly active edge sites, and interfacial states owing to the widths and edge configurations [2, 3, 4, 5]. These unique attributes hold significant potential for next‐generation nanoelectronics, quantum devices, and catalytic systems [6].

Among the various fabrication strategies of such nanostructures, molten salt‐assisted chemical vapor deposition (CVD) via a vapor–liquid–solid (VLS) growth mechanism has proven to be a reliable and scalable method for synthesizing uniform, large‐area TMDs with high crystalline quality [7, 8]. A distinctive feature of this VLS technique is the spontaneous formation of nano‐ to microribbons, typically several hundred nanometers to a few micrometers wide, directly on the basal plane of the 2D monolayers. This anisotropic growth arises from the reaction dynamics between alkali metal halides and metal oxide precursors, which yield molten intermediates that act as mobile solvents. These catalytic droplets migrate across the substrate surface, facilitating in‐plane material deposition and guiding the formation of ribbon‐like structures through localized supersaturation and directional solidification [7, 9, 10]. These “ribbons‐on‐monolayer” structures present a new avenue for engineering catalytic properties by modulating local charge redistribution, strain, and interfacial coupling [10, 11]. Despite their structural prominence, the catalytic influence of these microribbons on the underlying 2D monolayer remains poorly understood, particularly regarding their effect on electron transfer kinetics and basal plane activity. Addressing this challenge requires spatially resolved techniques capable of correlating nanoscale features with local electrochemical performance.

Scanning electrochemical cell microscopy (SECCM) is a powerful, high‐throughput technique for probing local electrochemical activity with nanoscale precision [12, 13]. By forming a confined nanodroplet as a mobile electrochemical cell via a nanopipette, SECCM enables steady‐state mass transport conditions and sensitive detection of Faradaic currents [14]. This technique has been instrumental in mapping heterogeneous activity in TMDs, revealing that defects such as edge sites, grain boundaries, and cracks exhibit significantly higher hydrogen evolution reaction (HER) performance than the comparatively inert basal plane, findings that align with density functional theory predictions favoring hydrogen adsorption at edge sites [15, 16, 17, 18, 19]. While most SECCM investigations have emphasized edge activity, emerging studies suggest that the basal plane also holds significant catalytic potential. For instance, Takahashi et al. demonstrated that electrochemical pre‐treatment can generate vacancies in MoS_2_ nanosheets, effectively activating the basal plane toward HER [20]. Their spatially resolved SECCM analysis revealed that only regions exposed to sufficiently strong electrochemical bias exhibited improved activity, characterized by increased current density, reduced overpotential, and enhanced kinetics. These effects were attributed to the formation of sulfur vacancies, which thermodynamically favor hydrogen adsorption over pristine lattice sites [21, 22, 23]. Furthermore, complementary findings by Schumacher et al. probed local HER activity across single and multilayer MoS_2_ [24]. Interestingly, they reported negligible differences in electrocatalytic performance between mono‐ and multilayer regions, suggesting that interlayer resistance plays a less significant role than factors such as polymeric residues or interfacial water layers trapped during the transfer process. Similar SECCM studies on MoS_2_ and WS_2_ heteronanosheets likewise found no significant variation in overpotentials or Tafel slopes with increasing layer number. Their observations attributed lateral heterogeneity in activity not to intrinsic electronic structure differences between layers, but to extrinsic factors affecting contact quality and ion transport [24]. In contrast, conventional electrochemical measurements often suggest a different picture. Yu et al. observed a systematic decrease in HER activity with increasing MoS_2_ thickness, with the exchange current density reduced by a factor of ∼4.5 for each additional layer [25, 26]. This decline was attributed to a vertical electron‐hopping barrier (0.119 V) that impedes charge transport through thicker films [26]. Supporting this interpretation, Zhuo et al. employed plasmonic imaging to show that monolayer MoS_2_ accumulates higher surface charge density, thereby enhancing conductivity and facilitating charge transfer at the electrolyte interface [27]. These results support the notion that thinner nanosheets perform better catalytically, largely due to the suppression of interlayer tunneling in thicker sheets. These contrasting findings suggest that the thickness dependence of HER activity in TMDs is not universal, but rather strongly influenced by methodology, interfacial quality, and environmental factors.

Despite these insights, a detailed understanding of basal plane heterogeneity at the nanoscale remains elusive, particularly in systems with hierarchical nanostructures such as microribbons atop a monolayer TMD. Given the inherently low edge‐to‐basal plane surface sites ratio in atomically thin TMDs, elucidating the spatial variability in basal plane activity is crucial for harnessing their full catalytic potential in electrochemical energy conversion.

In this work, we present the first high‐resolution SECCM study combined with correlative structural and spectroscopic techniques, optical microscopy, Raman spectroscopy, photoluminescence, transmission electron microscopy (TEM), atomic force microscopy (AFM), and kelvin probe force microscopy (KPFM) to directly probe how microribbons of varying thickness on WS_2_ monolayers regulate local HER activity. Our findings reveal that the exposed monolayer basal planes and edges significantly exhibit enhanced HER activity compared to microribbon regions. This variation is ascribed to layer‐dependent electron tunnelling and the strong interaction or doping effect of the underlying Au substrate. The microribbons, particularly the multilayer variants, demonstrate suppressed HER performance due to increased interactions between the multilayers and reduced charge transport efficiency. In contrast, single‐layer microribbons show higher activity than the multilayer regions, suggesting better electron accessibility due to a lower number of van der Waals barriers. These results provide a mechanistic understanding of how nanoscale structural features and interfacial effects modulate electrocatalytic behavior, offering valuable insights for the rational design of high‐performance TMD‐based catalysts through dimensional and morphological control.

## 2 Results and Discussion

### 2.1 Identification of Microribbons on Monolayer WS_2_

**FIGURE 1:**
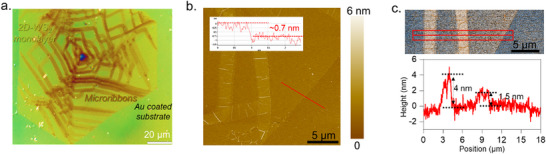
(a) An optical image of WS_2_ microribbons homoepitaxially grown on a 2D monolayer WS_2_ flake. (b) Atomic force microscopy topographic image highlighting the height profile of the WS_2_ monolayer. (c) High‐contrast AFM image obtained from (b) and height profile from the region marked by red rectangular regions, revealing variations in the thickness of microribbons.

The synthesis of microribbons‐on‐monolayer WS_2_ structures was achieved via molten salt‐assisted chemical vapor deposition (CVD) following a VLS growth mechanism [7, 8]. The precursor mixture comprised sodium tungstate dihydrate (Na_2_WO_4_·2H_2_O) and sodium chloride (NaCl), the latter serving as a flux and growth promoter with sulfur powder as the chalcogen source. Growth was conducted on thermally oxidized silicon substrates (SiO_2_/Si), as detailed in the methods section and schematically depicted in Figure S1. This synthetic strategy enables the homoepitaxial formation of microribbons with varying vertical thicknesses directly atop single‐crystalline WS_2_ monolayers, allowing precise control over interfacial morphology. An optical microscopy image of the resulting 2D material is displayed as Figure 1a, which shows the distinct edge geometry and in‐plane spatial heterogeneity of the microribbons relative to the underlying monolayer. These microribbons are displayed with uniform distribution across a 2D monolayer transferred on an Au‐coated SiO_2_/Si substrate. To further investigate the structural characteristics, AFM was employed. As shown in Figure 1b, the topographic scan confirms the monolayer nature of the WS_2_ base, exhibiting a thickness of approximately 0.7 nm, consistent with literature values of monolayer TMDs [28]. Superimposed on this basal plane are microribbons with a length of up to 120 µm, a width of 2–5 µm, and discrete thickness variations. The high‐contrast AFM image in Figure 1c reveals that the height of these vertical features ranges from ∼0.7 to ∼4 nm, indicating a monolayer to multilayer growth.

### 2.2 Catalytic Role of Surface Heterogeneities in As‐Grown WS_2_ and Au Effect

**FIGURE 2:**
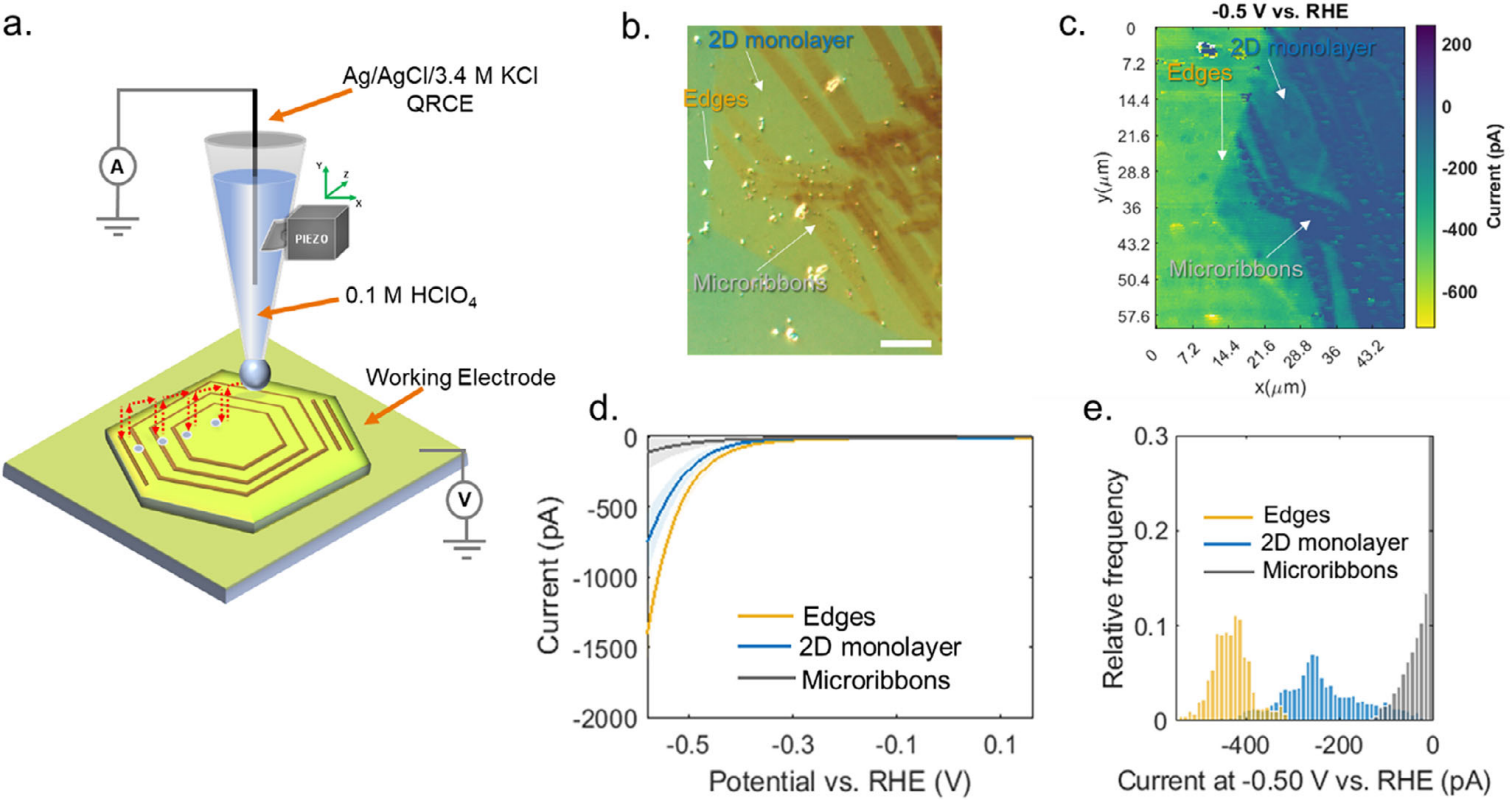
(a) Schematic illustration of the SECCM setup with WS_2_ transferred on an Au‐coated SiO_2_/Si substrate serving as the working electrode, 0.1m HClO_4_ as electrolyte, and a leakless Ag/AgCl (3.4 m KCl) electrode functioning as both the quasi‐reference and counter electrode (QRCE). (b) Optical image of the region investigated. Scale bar: 10 µm (c) SECCM current map acquired at −0.5 V vs RHE, revealing spatial variations in catalytic performance across three regions: the edges, basal plane of the 2D monolayer, and microribbons. (d) Averaged LSV curves calculated using the LSVs within the three distinct regions. (e) Histogram of current distributions for all pixels in each region, grouped according to Figure S4.

To identify the spatial catalytic variations across the as‐grown WS_2_ surface possessing a base 2D monolayer and microribbons, we first employed SECCM to probe the local electrocatalytic activity of the observed local heterogeneities. The SECCM setup is illustrated in Figure 2a. A single‐barrel nanopipette (∼54 nm inner diameter; Figure S2) filled with 0.1 m HClO_4_ was positioned above the WS_2_ surface, establishing a confined meniscus electrochemical cell. The WS_2_ sample transferred onto an Au‐coated SiO_2_/Si substrate served as the working electrode, while an Ag/AgCl (3.4 m KCl) leakless electrode inserted into the pipette functioned as both the quasi‐reference and counter electrode, enabling localized, two‐electrode electrochemical measurements (Figure S3). The region on the WS_2_ surface examined by the SECCM is shown as an optical microscope image in Figure 2b. All electrochemical potentials were referenced to the reversible hydrogen electrode (RHE), and measurements were conducted under an argon atmosphere. At each pixel location within the scanned grid (168 × 201 individual points), a linear sweep voltammogram (LSV) was acquired by performing SECCM in the so‐called hopping mode, allowing the construction of a spatial current map at −0.5 V vs RHE (Figure 2c). The resulting current map reveals three distinct regions of catalytic behavior, corresponding to the edges, basal planes of the 2D monolayer, and microribbons. A representative movie illustrating the spatial evolution of HER activity for the various regions as a function of potential is provided in the (Movie S1). Bright yellow pixels denote high‐activity regions, primarily at the edges, with cathodic currents ranging from −400 to −600 pA. In contrast, the basal planes of 2D monolayer (green pixels) exhibit moderate activity (−200 to −400 pA), while the microribbons (dark blue pixels) show substantially lower activity, with currents between 0 and −100 pA. Classification of the distinct electrocatalytic regions was performed through visual inspection of the current map acquired at −0.5 V vs RHE, identifying three representative domains based on their spatial morphology and electrochemical response.

Edge regions (shown in orange) demonstrated enhanced activity and appeared as distinct boundary zones, while the basal planes of the 2D monolayer (blue) showed higher current levels, and microribbons (grey) were revealed as low‐current responses. This spatial segmentation was implemented via pixel‐wise manual classification, as detailed in Figure S4. The averaged LSV from each region (Figure 2d) confirms this trend: edge sites consistently deliver the highest current densities, surpassing −1480 pA at −0.58 V vs RHE, approximately twice that of the basal plane of the 2D monolayer (−741 pA) and almost fifteen times higher than that of microribbons (−130 pA). It is worth mentioning that the enhancement in current density is not confined strictly to the geometric edge of the monolayer. A region extending roughly 2 µm from the edge also exhibits higher activity (Figure S4), which suggests that the electronic perturbations induced by undercoordinated S atoms at TMD edges propagate into the adjacent basal plane [29]. This extended region of enhanced activity is consistent with previous SECCM HER observations in 2H‐MoTe_2_, where local variations in electronic structure near edge defects increase the density of states and facilitate faster electron transfer even at nearby basal‐plane sites [30]. Based on the pipette size, these currents correspond to current densities of approximately −80.4 A cm^−2^ (edges), −38.6 A cm^−2^ (2D monolayer), and −6.6 A cm^−2^ (microribbons) as shown in Figure S5. The basal plane of the 2D monolayer shows more than 5‐fold higher current density than that of the microribbons. The observed high current density can be attributed to the electronic interaction with the underlying Au substrate, which plays a significant role in promoting rapid charge injection during localized electrochemical measurements [31]. Substrate‐induced enhancement of HER activity has been widely reported for atomically thin 2D catalysts, including TMDs and otherwise inert HER catalysts such as h‐BN. It is generally attributed to favorable orbital hybridization, improved electronic properties, and optimized hydrogen adsorption energetics by the underlying Au substrate [26, 31, 32]. To further quantify this spatial heterogeneity, a histogram of current densities at −0.5 V vs RHE for all measured pixels is presented in Figure 2e. The distribution underscores the dominant catalytic activity of the edge sites, with a pronounced peak beyond −450 pA. 2D monolayer exhibited a narrower distribution centered around −280 pA, while the microribbons are concentrated in the least active regime (0 to −80 pA), suggesting substantial suppression of HER activity in these regions.

### 2.3 Structural Characteristics of Microribbons on the Basal Plane

**FIGURE 3:**
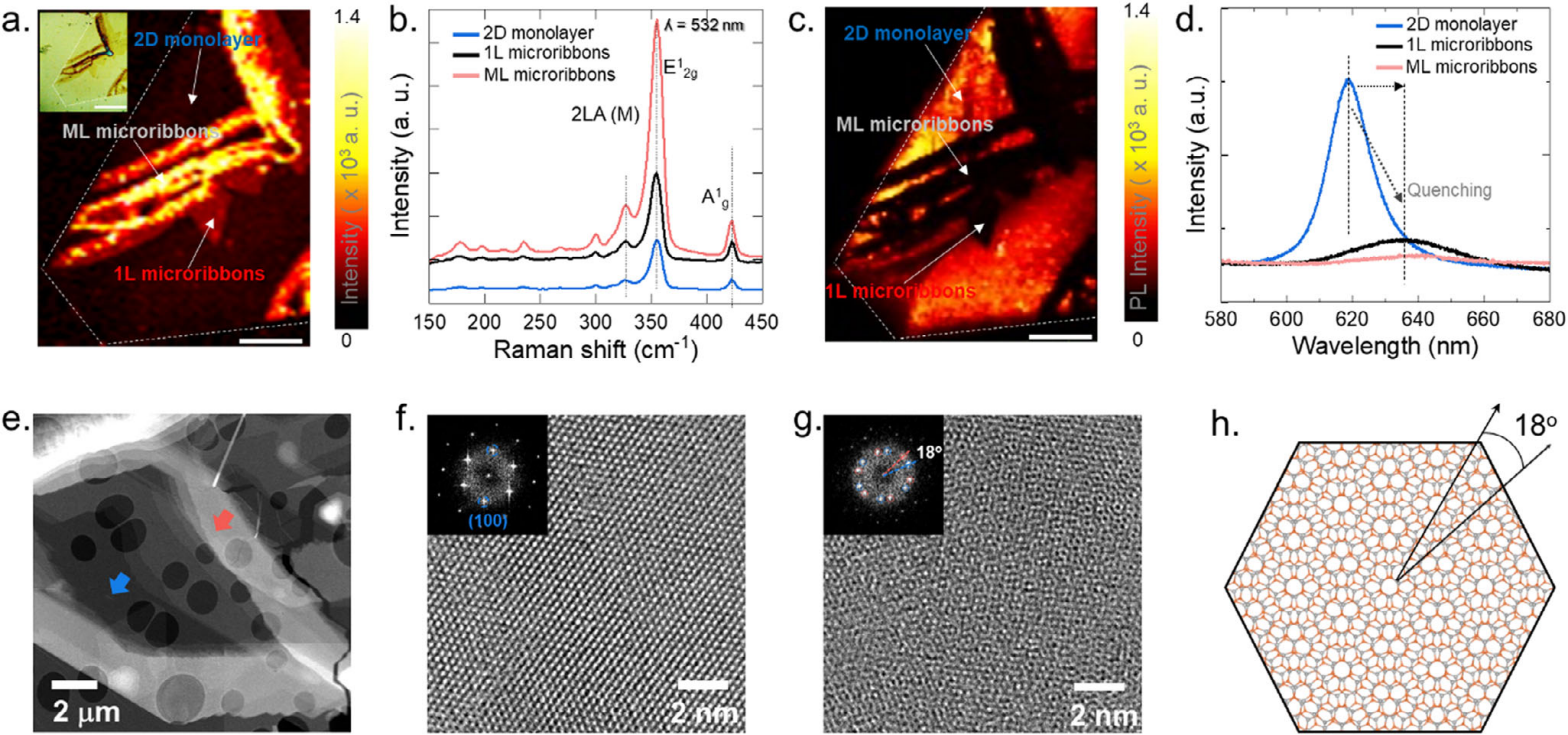
(a) Raman intensity map of WS_2_ microribbons on a monolayer flake taken at 353 cm^−1^. Inset: Optical image of the mapped region. (b) Raman spectra from marked regions in (a), showing vibrational differences between monolayer and microribbons. (c) Corresponding PL map recorded in the 616 – 632 nm range. (d) PL spectra from selected points in (c). (e) STEM image of both the basal plane of the monolayer (blue arrow) and microribbon (red arrow) regions. The circular features are due to the holey carbon‐coated support TEM grid. (f,g) HR‐TEM images of the monolayer and microribbon regions, respectively, with corresponding FFT patterns shown in the insets. (h) schematic representation of two stacked WS_2_ layers with an 18° misorientation angle. The scale bars in (a) and (c) are 20 µm.

We sought to understand the structural and electronic properties of microribbons formed on the monolayer WS_2_. A comprehensive spectroscopic characterization was conducted using Raman and photoluminescence (PL) spectroscopies at room temperature with 532 nm laser excitation. Two prominent Raman modes were observed: the out‐of‐plane A^1^_g_ mode and the second‐order longitudinal acoustic phonon at the M‐point [2LA(M)], which overlaps with the in‐plane E^1^_2g_ mode [28, 33]. Figure 3a shows Raman intensity mapping extracted at 353 cm^−1^, which shows the distribution of microribbons. The inset optical image identifies three regions: basal plane of the 2D monolayer, single‐layer (1L) microribbon, and multilayer (ML) microribbon. Raman intensity is highest in ML microribbon, followed by 1L microribbon, and lowest in the 2D monolayer, consistent with stacking‐induced enhancement in Raman scattering [34]. These findings are corroborated by AFM topography, which confirms increased thickness in the microribbon regions. Representative Raman spectra (Figure 3b) further emphasize the enhanced signal from microribbons, with subtle shifts and intensity variations due to strain and local thickness variations [28].

The enhanced scattering (high intensity) in 2LA(M), E^1^_2g,_ and A^1^_g_ modes in ribbon regions is attributed to multilayer stacking, since it is known that Raman intensity is mainly affected by the scattering volume, consistent with reported literature [28, 33]. Photoluminescence (PL) mapping was used to probe the local electronic structure. Figure 3c displays a PL intensity map in the A‐exciton range (616 – 635 nm), revealing the highest intensity in the 2D monolayer region, with a sharp quenching in the 1L and ML microribbons.

PL spectra (Figure 3d) show a redshift from ∼618 nm (monolayer) to ∼637 nm (microribbons), ascribed to thickness‐dependent excitonic behavior and interlayer coupling in stacked regions and the influence of the underlying substrate, which can induce charge transfer and modify the local strain state; factors known to shift exciton energies and quench emission intensity [10, 35]. The PL intensity in microribbons is four times lower than in the 2D monolayer, indicative of increased non‐radiative recombination pathways and transition from direct to indirect bandgap behavior in multilayers [33]. The A‐exciton peak of the 2D monolayer appears near 620 nm (∼2.0 eV), slightly blueshifted relative to the commonly reported position for as‐grown monolayer WS_2_ on SiO_2_ (∼1.92 eV) [35], which points to possible substrate‐induced charge doping or compressive strain by the underlying Au substrate. In contrast, the quenched PL peaks of the microribbons appear at 635 nm (∼1.95 eV) for the 1L ribbon and 640 nm (∼1.93 eV) for the ML ribbon, both showing pronounced redshifts. This gradual redshift indicates stronger interlayer coupling and increased trion formation (charged A excitons) in the stacked regions, which together lower the exciton energy and reduce PL emission.

To investigate the atomic structure of the WS_2_ domains, we utilized high‐resolution (scanning) transmission electron microscopy (HR‐(S)TEM). Figure 3e presents a STEM image of the WS_2_ sample containing both a 2D monolayer region (blue arrow) and a microribbon region (red arrow), corresponding to the morphological features previously identified in the optical and atomic force microscopy images (Figure 1). HR‐TEM images of the monolayer and microribbon, along with their respective fast Fourier transform (FFT) patterns shown in Figure 3f,g, reveal high crystallinity in both regions. The FFT of the monolayer (Figure 3f) displays a well‐ordered hexagonal lattice, typically for the basal plane of 2H‐WS_2_ viewed in a perpendicular direction. The visible interplanar spacing in the HRTEM images is measured to be 0.27 nm, characteristic of the {100} planes of 2H‐WS_2_ [36]. In contrast, the microribbon region (Figure 3g) exhibits a distinct moiré pattern, arising from the superposition of two misoriented WS_2_ layers. The FFT inset reveals two sets of diffraction spots (highlighted by blue and red circles), corresponding to individual layers with a measured rotational misalignment of 18°, confirming the presence of two vertically stacked layers with differing in‐plane orientations in the microribbon region, i.e., the presence of 1L microribbon. A schematic illustration in Figure 3h depicts the stacking configuration of two WS_2_ layers with an 18° in‐plane misorientation, leading to the observed moiré pattern consistent with the experimental results in Figure 3g.

### 2.4 Correlated SECCM with Raman and Photoluminescence Mapping

**FIGURE 4:**
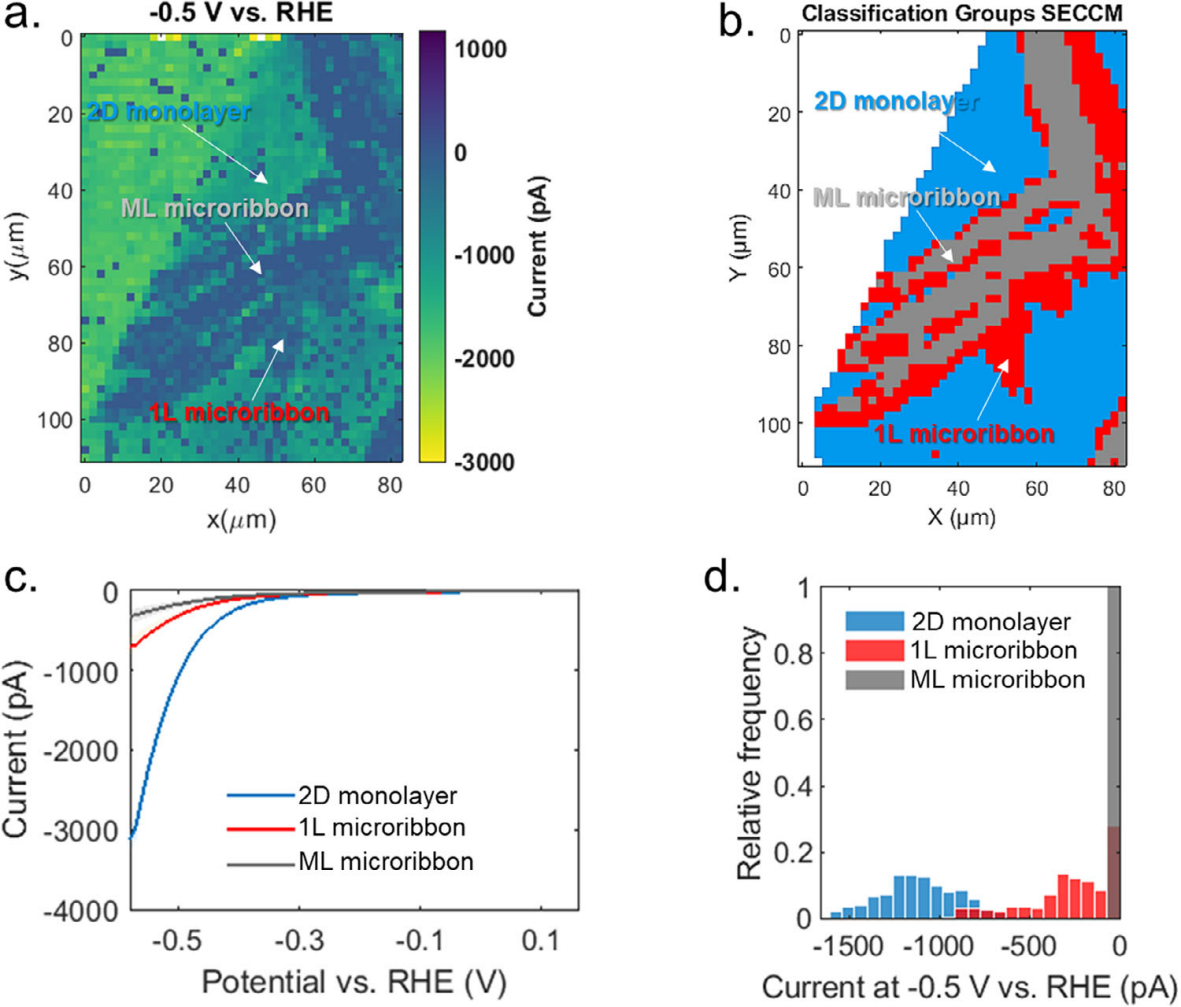
(a) SECCM current map acquired at −0.5 V vs RHE. (b) Classified electrochemical activity regions based on correlation with Raman mapping, with a uniform grid spacing of 2 µm along both axes. (c) LSV curves obtained by averaging the LSVs recorded over different measuring areas over the three distinct regions: the 2D monolayer, single‐layer (1L) microribbon, and multilayer (ML) microribbon. (d) Histogram of current distributions for all pixels within each region, providing a statistical comparison of their electrocatalytic activity.

After unravelling the structural and electronic characteristics of the microribbons, we then examined how the different layer stacking of these features influences electrocatalytic performance. A correlative analysis integrating spatially resolved Raman spectroscopy with SECCM measurements to map local HER activity with structural specificity was done. As a selection criterion, the intensity of the E^1^_2g_ Raman band was used, and three groups were established (Figure S6). Figure 4a presents the spatial current distribution (66 × 56 individual points) recorded at −0.5 V vs RHE using a larger pipette (∼118 nm inner diameter), depicting electrocatalytic differences among the three regions. The basal plane of the 2D monolayer (yellow in color scale) exhibits the highest catalytic activity, with localized currents reaching up to −3000 pA. In contrast, 1L microribbon shows intermediate activity, with currents ranging between −500 and −750 pA, while ML microribbons display minimal activity, with currents barely reaching −300 pA. A representative movie illustrating the spatial evolution of HER activity as a function of potential is provided in the (Movie S2). To correlate local structure with the electrochemical activity, Raman classification was mapped onto the SECCM current grid. Raman and SECCM maps were cropped to a common ∼82 × 110 µm region, and grid alignment was achieved via 2D interpolation. The Raman grid (1.5 × 1.6 µm spacing) was interpolated onto the SECCM grid (2 µm spacing) using a nearest‐neighbor method [37] (see Figure S7 for details).

Based on our interpolation of the electrochemical current map and correlative Raman mapping, classification of the group regions was determined in Figure 4b. The electrochemical trends are further illustrated in the averaged LSVs shown in Figure 4c.

The LSV corresponding to the basal plane of the 2D monolayer (blue curve) exhibits over five times higher current response than the 1L microribbon (red curve), while the ML microribbon (grey curve) yields negligible current, indicating severely suppressed catalytic performance. At −0.58 V vs RHE, the basal plane reached −3200 pA, compared to −635 and −330 pA for the 1L and ML microribbons, respectively. Normalizing these currents by the pipette size yields current densities of approximately −25.2 A cm^−2^ (2D monolayer),−4.3 A cm^−2^ (1L microribbon), and −2.3 A cm^−2^ (ML microribbon) as shown in Figure S8. Accordingly, the basal plane demonstrates more than 5‐fold and 10‐fold higher current densities than the 1L and ML microribbons, respectively. This trend is consistent across measurements with different pipette sizes (Figures S4 and S8), affirming a persistent trend in catalytic performance: basal plane of 2D monolayer > 1L microribbon > ML microribbon. Figure 4d summarizes the statistical distribution of HER currents across each region. The histogram confirms a clear stratification in performance: basal planes dominate the high‐activity range, 1L microribbons populate the intermediate regime, and ML microribbons cluster near zero current. This analysis emphasizes the strong influence of microribbon thickness on catalytic heterogeneities in the basal plane of the 2D monolayer. Specifically, while 1L microribbons retain moderate HER activity, ML microribbons become increasingly inactive.

Further insight into the relationship between local structure and electrochemical activity was obtained through correlative analysis of Raman, PL, and SECCM data (see experimental section for details). Previous studies have shown that spatial variations in PL and Raman responses are correlated with local strain, doping, and defect‐induced inhomogeneities in WS_2_ monolayers [38]. As shown in Figures S9 and S10, correlating the Raman (A_1g_ and E^1^_2g_) and PL intensities with the current at −0.5 V vs RHE reveals a clear thickness‐dependent trend. ML microribbons show the highest Raman intensities and lowest PL intensities but generate negligible current, confirming their poor catalytic activity. The 1L microribbons display intermediate values for both PL intensity and current. The 2D monolayer regions show the strongest catalytic response (currents up to −1500 pA), correlating with very narrow distribution of lower Raman intensities and with a much broader distribution of PL intensities. Overall, regions with weaker Raman modes typically indicate thinner or structurally perturbed areas that interact more strongly with the Au substrate, exhibit higher activity, consistent with enhanced defect density, local strain, and substrate‐induced doping.

**FIGURE 5:**
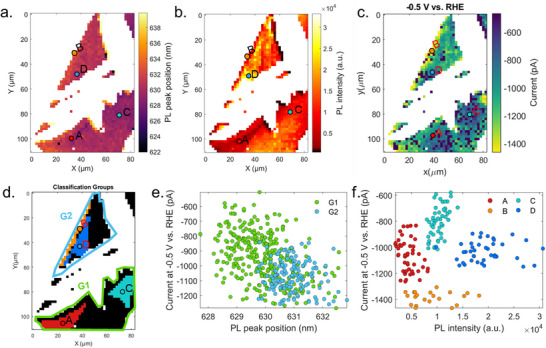
(a) PL peak position. (b) PL intensity and (c) SECCM current recorded at −0.5 V vs RHE maps of the monolayer regions. (d) A group of SECCM points located on the monolayer that are in the two groups discussed in Figure S11a. Correlation of the SECCM currents recorded at −0.5 V vs RHE with the PL peak position, with (e) the points grouped in Figure S11 and (f) those marked in Figure 5d.

To resolve spatial heterogeneity within the monolayer WS_2_, the selected regions within the monolayer flake were divided into two groups, G1 and G2, that correspond to the lower and upper parts of the monolayer, respectively, and are separated by the microribbons. In these two groups, two regions were further selected: in G1, regions A and C, and in G2, regions B and D, based on the PL intensity (Figure 5b). These groups are also located on different parts of the monolayer and help distinguish how strain, defects, and edge‐induced perturbations influence optical behavior and catalytic activity. Figure 5 summarizes the trends observed exclusively within the monolayer domains.

The PL peak position (Figure 5a), PL intensity (Figure 5b), SECCM current (Figure 5c), and a schematic illustration of the various groupings in Figure 5d show the systematic variation across the flake. Plotting current versus PL peak position, which is modulated by tensile strain and defect‐induced excitonic shifts, enhances charge transfer [38] (Figure 5e) and yields a clear linear trend. This observation is also supported by the linear correlation between SECCM current and Raman peak intensities; inverse linear correlations are observed for the local currents with the A_1g_ (Figure S11b) and E^1^_2g_ (Figure S11c) intensities that are sensitive to charge‐trapping defects and local strain, respectively. This indicates that local strained regions, as well as defects present in the monolayer, impact the electrocatalytic activity. The decrease in the HER current with the increase in the E^1^_2g_ intensity (Figure S11c–e) indicates that excessive strain coupled to structural defects disrupts the lattice and suppresses charge‐transfer kinetics, thereby reducing HER activity [39].

The changes in the PL intensity over the WS_2_ monolayer, visualized in Figure 5b, further differentiate structural domains. From the current vs PL intensity plot in Figure 5f, regions with low PL intensity (A and B) show higher activity, consistent with quenching by strain or defects. In contrast, regions with higher PL intensity (C and D) show reduced activity, characteristic of more crystalline, low‐defect areas. A qualitative analysis and comparison of the local electrocatalytic activity, Raman, and PL characteristic signals over the regions A, B, C, and D is summarized in Table 1. Overall, the highest local electrocatalytic activity is recorded in region B, at the WS_2_ edges, where the highest defect density and tensile strain are present. On the contrary, the lowest electrocatalytic activity is recorded in region C, which is located deeper inside the basal plane, a region with low strain and fewer defects. The moderate local electrocatalytic activities are recorded on regions D and A. Region D, adjacent to the edge (B), shows higher PL intensity yet strongly PL redshifted than C, indicating its crystalline domain is affected by tensile strain extending inward from the edge, which can boost the electrocatalytic activity compared to the inside regions, such as C. The A region, which extends from the edge toward the interior of the WS_2_ monolayer, exhibits a mixed domain governed by localized strain gradients, vacancy formation, and partial edge influence, resulting in medium electrocatalytic activity. Together, these observations demonstrate that strain‐ and defect‐modulated electronic environments play a decisive role in governing the HER performance of 2D monolayer WS_2_ at the nanoscale.

**TABLE 1:** Summary of region‐specific structural, optical, and electrochemical characteristics within monolayer WS_2_ regions.

Region	Location	PL intensity	Raman A_1g_ intensity	Raman E^1^_2g_ intensity	SECCM current	Interpretation
B (G2)	Physical edge	Moderate and broad	Lowest	Lowest	Highest	Edge‐dominated region with high defect density and tensile strain; strongest catalytic activity.
D (G2)	Inner domain adjacent to the edge	Highest and broad	Moderate	Moderate	Moderate	Crystalline region influenced by edge‐induced strain; higher current despite high PL intensity.
C (G1)	Inner basal‐plane region	Moderate	Highest	Highest	Lowest	Region with low strain and fewer defects; intrinsic monolayer behavior and lowest activity.
A (G1)	Local strain/defects; partly edge‐affected	Lowest	Moderate	Moderate	Moderate	Structurally heterogeneous; influenced by strain, vacancies, and partial edge proximity.

To probe the effect of electrolyte conditions, we performed additional measurements in a slightly higher‐pH electrolyte (∼3, 0.001 m HClO_4_ + 0.1 m NaClO_4_), as shown in Figure S12. Consistent with the trends observed at pH ∼ 1, the relative activity taken at ‐0.5 V vs RHE remained unchanged: ML microribbons exhibited negligible current, 1L microribbons reached −30 pA, and the 2D monolayer showed the highest activity at −45 pA. The overall decrease in current compared to pH ∼ 1 can be attributed to the lower proton concentration, which directly reduces the rate of proton‐coupled electron transfer at the electrode surface, which limits the HER rate. The thickness‐dependent reduction in HER activity trend parallels earlier observations by Yu et al., who demonstrated a 4‐fold decrease in HER activity per additional layer in MoS_2_, attributing the decline to increased interlayer potential barriers and reduced vertical electron transport efficiency in the multilayer stack [25, 40]. In contrast, our measurements provide the first direct experimental visualization of localized heterogeneities, clearly revealing the reduced catalytic performance of multiple‐layered microribbons on a monolayer TMD.

### 2.5 Origin of the Reduced Catalytic Activity on Microribbons

To further clarify the influence of microribbon thickness on HER activity, we employed KPFM as a tool to probe the local electronic properties of WS_2_ domains. KPFM provides nanoscale mapping of surface potential and work function, parameters that are closely related to the ease of electron transfer. Since efficient electron transfer is a key step in HER, these results can be considered an experimental analogue to hydrogen adsorption energetics [41]. In this way, the variation in surface potential with microribbon thickness directly supports the observed inhibition in HER activity.

The measurements were performed using the Park NX10 AFM instrument to acquire topography, surface potential, and work function maps of the WS_2_ monolayers and microribbons under ambient conditions (see experimental section for details). KPFM quantitatively maps the contact potential difference (CPD) with nanometer‐scale resolution, providing a direct link between local electronic structure and catalytic performance. The CPD is defined as:

(1) $$V_{\mathrm{CPD}}=\frac{\phi_{sample}-\phi_{tip}}{q}$$

where ϕ_sample_ and ϕ_tip_ are the work function of the sample and the AFM probe, respectively. During calibration, the tip's effective work function was determined against a gold reference substrate (ϕ_Au_ ∼ 5.12eV), consistent with previous literature [42] ensuring quantitative reliability of the extracted work function values. V_CPD_ is obtained when the applied DC bias compensates the electrostatic interaction, allowing conversion of the measured CPD into absolute surface potential and work function values.

**FIGURE 6:**
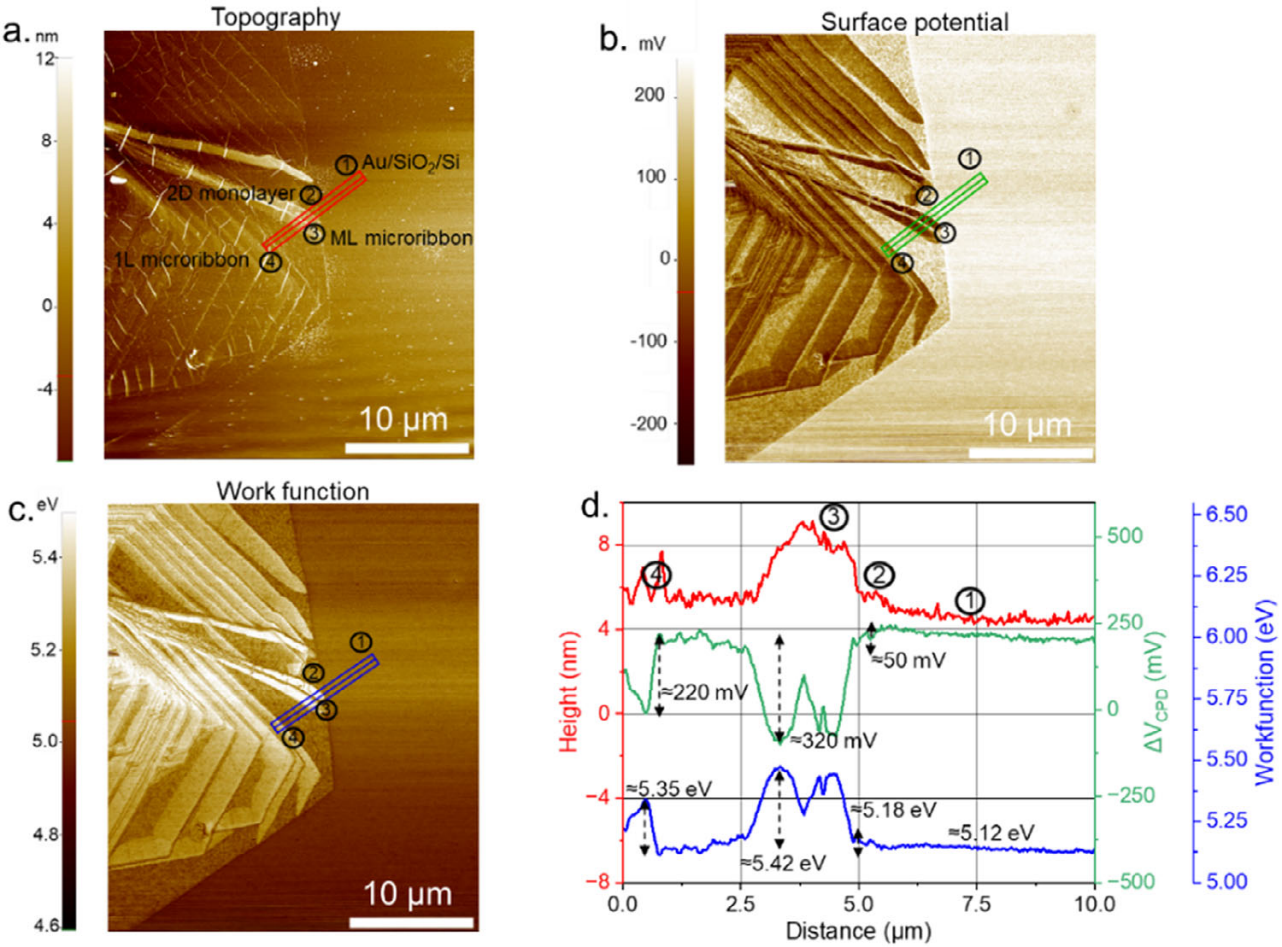
(a) AFM topography (b) corresponding to the KPFM surface potential map. (c) work function map derived from the CPD values for a WS_2_ region containing the substrate (1), 2D monolayer WS_2_ (2), 1L microribbons (3), and ML microribbons (4). (d) Profiles extracted along the lines indicated in (a–c), respectively: AFM height profile (red), surface potential (green), and work function (blue).

Figure 6 shows AFM topography and KPFM images of WS_2_ microribbons of varying thickness on a monolayer supported by an Au/SiO_2_/Si substrate. AFM topography (Figure 6a) confirms the presence of 1L to ML microribbons with the underlying 2D monolayer WS_2_. The KPFM map (Figure 6b) reveals a thickness‐dependent contrast: 2D monolayer regions show a potential shift of ∼ −50 mV, 1L microribbons ∼ −220 mV, and ML microribbons ∼ −320 mV relative to the tip. Line profiles extracted across the regions (Figure 6d) further show the systematic decrease in CPD with increasing layer number. This trend is consistent with earlier reports on MoS_2_/Au systems, where the surface potential difference diminishes with thickness due to interface charge screening. Previous studies demonstrated that the difference between the surface potential of MoS_2_ flakes and that of the bulk (ΔV*_CPD_* = V*_surf_* − V*_bulk_*) decreases with layer number, with ΔV_CPD_ gradually approaching zero for thicker flakes [42, 43]. The corresponding work function map derived from the CPD values is shown in Figure 6c. The estimated work functions were ∼5.18 eV for the 2D monolayer, ∼5.35 eV for the 1L microribbons, and ∼5.45 eV for the ML microribbons, all slightly larger than the Au reference (5.12 eV). Within each WS_2_ domain, the work function distribution remained relatively homogeneous. The observed steady increase in work function with thickness reflects the combined effects of WS_2_ band structure evolution and substrate screening: monolayer WS_2_ has a direct bandgap and relatively high Fermi level, resulting in a smaller work function, whereas thicker WS_2_ approaches the indirect bandgap of bulk, lowering the Fermi level and increasing the work function. Charge transfer from the Au substrate reduces the work function of ultrathin layers, but this influence is increasingly screened in thicker WS_2_. Notably, the lower work function of monolayer regions suggests enhanced interfacial electron transfer, which is consistent with previous reports for other complex catalysts [44, 45] thus, it contributes to higher HER activity in thinner WS_2_ domains, unlike those in multilayer domains.

**FIGURE 7:**
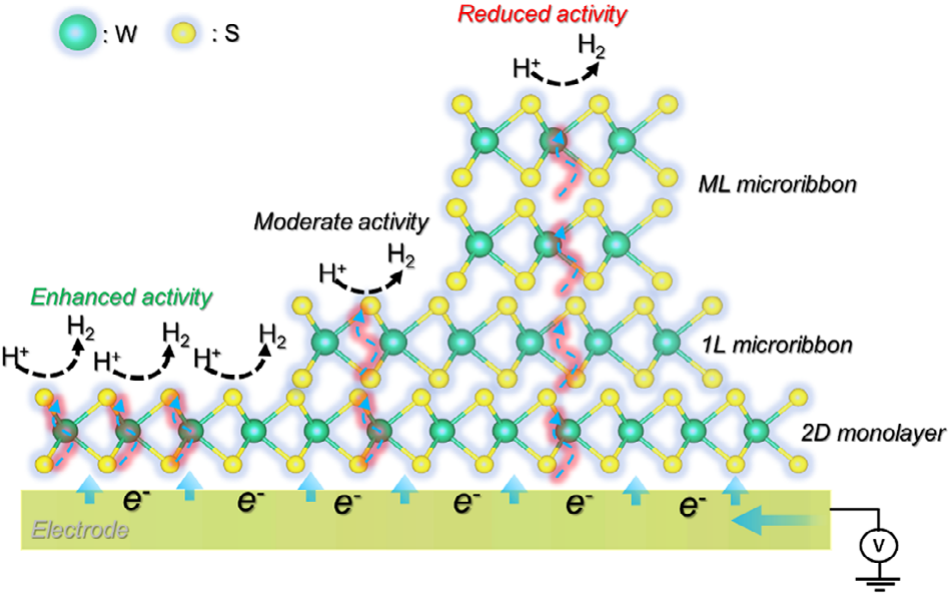
Schematic illustration of the influence of 2D monolayer and microribbon thickness on vertical charge transport and HER activity.

The structural variations in the microribbons observed in Raman mapping, (S)TEM, AFM, and KPFM images, with spatially resolved SECCM activity maps, enabled a clear thickness‐dependent trend in HER activity to be established. This confirms the critical role of vertical charge transport in governing catalytic efficiency across the various domains in our catalyst. We propose that the observed decline in HER performance with increasing microribbon thickness originates from limitations in interlayer electron transport. Efficient hydrogen evolution on WS_2_ requires that electrons are transferred from the substrate through the structures and delivered to catalytically active sites on the surface. In the 2D monolayer WS_2_, this transport pathway is direct and minimally resistive. However, as the number of layers increases in microribbons, charge carriers must tunnel through multiple van der Waals‐bonded interfaces, resulting in an energetically unfavorable process. As illustrated in Figure 7, under sufficient reductive bias, the 2D monolayer regions accumulate charge more effectively, facilitating rapid electron transfer to surface‐adsorbed protons for HER. In contrast, the thicker microribbons suffer from poor interfacial coupling and interlayer charge screening, which hinder efficient electron delivery to the active sites. This effect is obvious in ML microribbons, which must overcome a significantly larger tunnelling barrier, leading to their markedly lower activity.

## 3 Conclusion

We investigated the intricate structure–activity relationships governing HER in microribbons on 2D monolayer WS_2_. Using SECCM in combination with spectroscopy and microscopy techniques, we confirm that edge sites exhibited the highest catalytic activity, consistent with previous observations. Among the basal regions, the 2D monolayer WS_2_ showed the next highest performance, achieving current densities up to −25.2 A cm^−2^ at −0.58 V vs RHE. The result showed a ∼5‐fold increase than those of homoepitaxially grown 1L microribbons (−4.3 A cm^−2^), over 10‐fold increase than ML microribbons (−2.3 A cm^−2^). The enhancement is attributed to enhanced charge transfer from the substrate that effectively tunes the electronic properties of the basal plane of the 2D monolayer. In contrast, microribbons, particularly those with multilayer stacking, showed reduced catalytic performance due to interlayer van der Waals coupling and poor charge transport, which limits the accessibility of electrons to active sites. However, single‐layer microribbons retain moderate activity, underscoring the importance of minimizing thickness to preserve electrochemical reactivity. These findings emphasize the uniqueness of SECCM combined with correlational PL/Raman mapping to probe nanoscale catalytic behavior, identify defects, and reveal their influence on local activity. By leveraging dimensional control and interfacial engineering, this work offers a rational strategy for optimizing TMD‐based materials toward efficient, scalable hydrogen production.

## 4 Experimental Section

### 4.1 Growth of Microribbons on Monolayer WS_2_

Microribbons on monolayer WS_2_ were synthesized using a molten salt‐assisted chemical vapor deposition (CVD) method. The liquid precursor was prepared by dissolving sodium tungstate dihydrate (Na_2_WO_4_·2H_2_O, ≥99%, Sigma–Aldrich) and sodium chloride (NaCl) in the ratio 5:1 in deionized water to yield a 0.05 m Na_2_WO_4_ solution. This precursor solution was spin‐coated onto UV‐O_3_‐treated SiO_2_/Si substrates at 3000 rpm for 60 s to ensure uniform coverage. The coated substrates were then placed at the center of a 2‐inch quartz tube furnace. 500 mg of sulfur powder, loaded in a quartz crucible, was positioned upstream to serve as the sulfur source. The CVD chamber was initially evacuated to ∼1 Pa and subsequently backfilled to atmospheric pressure using Ar gas (350 sccm) or a forming gas mixture (Ar with 5 vol.% H_2_ at 100 sccm), depending on the configuration [46]. The system was then ramped to 800 °C at a rate of 15 °C/min. To facilitate sulfurization and crystal growth, sulfur vapor sublimated at ∼200 °C was carried downstream, enabling the formation of vertically stacked microribbon structures atop the monolayer WS_2_. The possible equation for the reaction is proposed as:

(2) $$\begin{array}{ccc} Na_{2}WO_{4}\cdot 2H_{2} & & O+2\mathrm{NaCl}+3S\;\to WS_{2}\left(s\right)+2Na_{2}SO_{4}+Cl_{2}\;(g)\\ & & \;+2H_{2}O\;\left(g\right) \end{array}$$

After the reaction, the temperature was turned off, and the furnace was allowed to cool naturally to room temperature under an inert Ar atmosphere. For characterization and SECCM measurements, the synthesized WS_2_ films were transferred using a PMMA‐assisted wet transfer method [46]. A PMMA film was spin‐coated on the as‐grown WS_2_/SiO_2_/Si sample, and the underlying substrate was etched away in 35 wt.% KOH solution. The PMMA/WS_2_ was rinsed in deionized water three times, then transferred onto Au‐coated SiO_2_/Si substrates or a TEM grid of the carbon perforated film on copper mesh (Plano, 400 mesh, copper). After drying under ∼20% relative humidity at room temperature for 2 h, the PMMA was removed by sequential rinsing in acetone and isopropanol (IPA). For SECCM measurements, the Au/SiO_2_/Si‐supported samples were annealed in forming gas (Ar/H_2_) at 300 °C for 3 h to remove any residual polymer contaminants.

### 4.2 Material Characterization:

#### 4.2.1 Raman and Photoluminescence (PL) Spectroscopy

The morphology and spatial distribution of microribbons on monolayer WS_2_ were first examined by polarised optical microscopy (Nikon LV‐IM, Nikon). Raman and photoluminescence (PL) spectra were acquired using a confocal Raman microscope (inVia, Renishaw) equipped with a 532 nm excitation laser and a 50× objective lens. A diffraction grating with 1800 grooves/mm was used to disperse the signal, which was detected using an electron‐multiplying charge‐coupled device (CCD, Andor). The laser spot diameter was ∼1 µm. The 520 cm^−1^ phonon mode from the silicon substrate was used for calibration. All spectra were collected on Au‐coated SiO_2_/Si substrates. Data processing, including baseline correction, normalization, and peak fitting, was performed using the Wire software (Renishaw) and OriginPro.

#### 4.2.2 Atomic Force Microscopy (AFM)

AFM topography was acquired in non‐contact mode with a PPP‐NCHR cantilever (resonance frequency ∼300 kHz; spring constant ∼42 N/m) using the Park NX10 AFM instrument. Scans were recorded at 512 × 512 resolution at a scan speed of 0.2–0.3 Hz with a tapping amplitude of 16 nm. Data processing and topographical analysis were performed using Park Systems’ XEI software. Surface topography maps were plane‐corrected and drift‐compensated. Cross‐sectional analysis was used to quantify local topographical heterogeneity across the microribbon‐on‐monolayer structures.

#### 4.2.3 Kelvin Probe Force Microscopy (KPFM)

KPFM images were acquired in non‐contact electrostatic force microscopy (EFM) mode using a PPP‐EFM cantilever (Park Systems NX10). The Cr–Pt/Ir‐coated probe (tip radius ∼25 nm; tip length ∼15 µm; spring constant ∼2.8 N m^−^^1^) was operated in non‐contact mode, allowing simultaneous acquisition of surface topography and surface potential. During KPFM operation, an AC modulation voltage of ≈1.0 V was applied at the cantilever resonance frequency (∼79.1 kHz). A DC bias (initially set to 0 V) was dynamically adjusted by the feedback loop to nullify the electrostatic force component at the modulation frequency, with the compensated DC bias corresponding to the local contact potential difference (CPD). The tip work function was calibrated against a gold reference (ϕAu ∼5.12 eV) before measurements. Images of topography, surface potential, and work function were collected with 1024 pixels per line at a scan rate of 0.1 Hz and oscillation amplitude of ∼31 nm.

#### 4.2.4 (Scanning) Transmission Electron Microscopy ((S)TEM)

High‐resolution (scanning) transmission electron microscopy was carried out using a Cs‐image corrected Titan Themis 80–300 from Thermo Fisher operated at 300 kV. The HR‐TEM images using parallel illumination were acquired with a 4kx4k CCD camera. Together with the FFT of the images, the atomic structure of the 2D monolayer and microribbon regions was investigated. In addition, low‐magnification high‐angle annular dark field STEM images were taken with a convergent beam and an annular detector to determine the overall morphology.

#### 4.2.5 Scanning Electrochemical Cell Microscopy (SECCM)

Electrochemical measurements were conducted using a home‐built SECCM platform used in our previous publication [24] (see Figure S3 for details). The setup was controlled via a field‐programmable gate array (FPGA) card (USB‐7855R, National Instruments) and a customized LabVIEW program adapted from the Warwick Electrochemical Scanning Probe Microscopy (WEC‐SPM) toolbox. The coarse positioning of the sample was achieved using x, y, z stepper motors (Owis) and a Lang LStep PCIe controller, allowing selection of regions of interest on the substrate surface. The tip–sample distance (∼50 µm) was adjusted with the aid of a camera (Motic) equipped with a VZM 200i zoom lens (Edmund Optics) and an EUROMEX LED cold light source for illumination.

High‐resolution scanning was performed in hopping mode using a piezoelectric positioning system (x, y, z piezo cube; C3.100, PIEZOCONCEPT) with its corresponding controller, enabling fine movement of the pipette holder. During each SECCM measurement, an electrochemical cell was formed at the point of contact between the hanging droplet at the nanopipette tip and the substrate surface (WS_2_/Au_SiO_2_/Si). A two‐electrode configuration was employed, where the working electrode was the substrate and the QRCE was a leakless Ag/AgCl electrode (3.4 m KCl, Innovative Instruments) inserted into the nanopipette filled with 0.1 m HClO_4_. The pipette tip opening ranged around 54 to 118 nm. The open circuit potential (OCP) of the quasi‐reference counter electrode (QRCE) was measured against a standard Ag/AgCl/3 m KCl reference electrode before SECCM experiments. The nanopipette filled with electrolyte and the Ag/AgCl/3.4 m KCl electrode were both immersed in a one‐compartment electrochemical cell containing 0.1 m HClO_4_. To convert the potentials recorded during SECCM measurements from the Ag/AgCl QRCE scale to the reversible hydrogen electrode (RHE) scale, the following equation was used:

(3) $$E_{\mathrm{RHE}\;}[V]=E_{\mathrm{Ag}/\mathrm{AgCl}}+0.210+E_{\mathrm{OCP}\;}+0.0592\;\times \;\mathrm{pH}$$

where E_Ag/AgCl_ was the applied potential versus the quasi‐reference counter electrode (QRCE), 0.210 V was the standard potential of the Ag/AgCl/3 m KCl reference electrode at 25 °C, and E_OCP_ was the measured open circuit potential difference between the QRCE and the Ag/AgCl/3 m KCl reference electrode.

High‐resolution spatially resolved electrochemical maps were obtained with the described setup. For Figure 2, measurements were performed over 168 × 201 individual spots with a lateral step size of 300 nm, covering an area of 50.1 × 60 µm. For Figure 4, we measured over 66 × 56 points with a lateral step of 2 µm, generating a 130 × 110 µm map.

#### 4.2.6 Processing and Reporting SECCM Data

The raw.tdms files obtained after SECCM scans were entirely processed using a MATLAB (MathWorks, USA) script. The scripts enable the extraction of all recorded data and their assignment to the recorded data to the spot from which they were collected. The potential was converted from Ag/AgCl (3.4 m KCl) scale to RHE scale, the current was converted from A to pA, and normalized with the tip size for the figures where current density was reported. The voltammetry signals were processed to produce heatmaps and videos by extracting the current value at each applied potential.

The Raman and PL data in each spot were imported and processed in MATLAB to calculate the maximum intensity and the position of the corresponding peak by using the built‐in max function in a restricted interval (i.e., 325–375 cm^−1^ for *E^1^_2g_*, and 400–430 cm^−1^ for A*_1g_*), which gives the value and position of the peak. With this data, the corresponding maps were calculated.

In order to produce grouped histograms and scatter plots, the data was grouped in different ways; the grouping strategy (i.e., interactive selection, clustering algorithms) was explained in detail in the corresponding section/figure. The constructed groups and the data used for the current maps and Raman or PL intensity maps were used to plot the grouped histogram and scatter plots displayed in the text.

### 4.3 Statistical Analysis

For the generation of averaged LSV, histograms, and scatter plots, an initial filtering of data was done. Where data points deviating by more than 1.5 standard deviations from the group median were considered outliers. These values were omitted from all subsequent calculations. The statistical parameters reported, including mean values and confidence intervals, were therefore based solely on the filtered datasets. For each measurement, the mean current density ($\bar{x}$) and the 95% confidence interval (= 1.96 × Standard Error of the Mean). All the scripts used for processing were provided online along with the dataset as additional information.

## Conflicts of Interest

The authors declare no conflict of interest.

## Supporting information




**Supporting File 1**: smll72108‐sup‐0001‐SuppMat.docx

**Supporting File 2**: smll72108‐sup‐0002‐MovieS1.mp4

**Supporting File 3**: smll72108‐sup‐0003‐MovieS2.mp4

## Data Availability

The data that support the findings of this study are openly available at Zenodo at https://doi.org/10.5281/zenodo.17733854.
