# Supplementary material for: Impact of Structural Heterogeneities in Hierarchically Grown Microribbons on 2D WS2 Monolayer on the Measured Local Hydrogen Evolution Activity on Au Substrate
Source: Small. 2026 Jan 8;22(10):e10517. doi: 10.1002/smll.202510517 (PMC12910432; doi:10.1002/smll.202510517)
Supplement: Supplementary file 1 — Supporting File 1: smll72108‐sup‐0001‐SuppMat.docx [file SMLL-22-e10517-s002.docx]

Supporting Information

**Impact of structural heterogeneities in hierarchically grown microribbons on 2D WS_2_ monolayer on the measured local hydrogen evolution activity on Au substrate**

*Laud Anim Adofo, Alejandro E. Pérez Mendoza,* [*Miran Joo*](https://www.mpie.de/person/137892/2903079)*, Lithin Madayan-Banatheth, Andrew Ben-Smith, Ki Kang Kim, Christina Scheu, and Corina Andronescu*^*^

Dr. L. A. Adofo, Dr. A. E. Perez-Mendoza, L. Madayan-Banatheth, Prof. Dr. C. Andronescu
Chemical Technology III, University of Duisburg-Essen, Carl-Benz-Straße 199, 47057 Duisburg, Germany

Dr. M. Joo, Prof. C. Scheu

Nanoanalytics and Interfaces, Max Planck Institute for Sustainable Materials, 40237 Düsseldorf, Germany

Dr. A. B. -Smith, Prof. K. K. Kim

Department of Energy Science, Sungkyunkwan University, Suwon, 16419, Republic of Korea

Prof. Dr. C. Andronescu

Center for Nanointegration University of Duisburg-Essen (CENIDE) Carl-Benz-Straße 199, 47057 Duisburg, Germany

E-mail: [corina.andronescu@uni-due.de](mailto:corina.andronescu@uni-due.de)


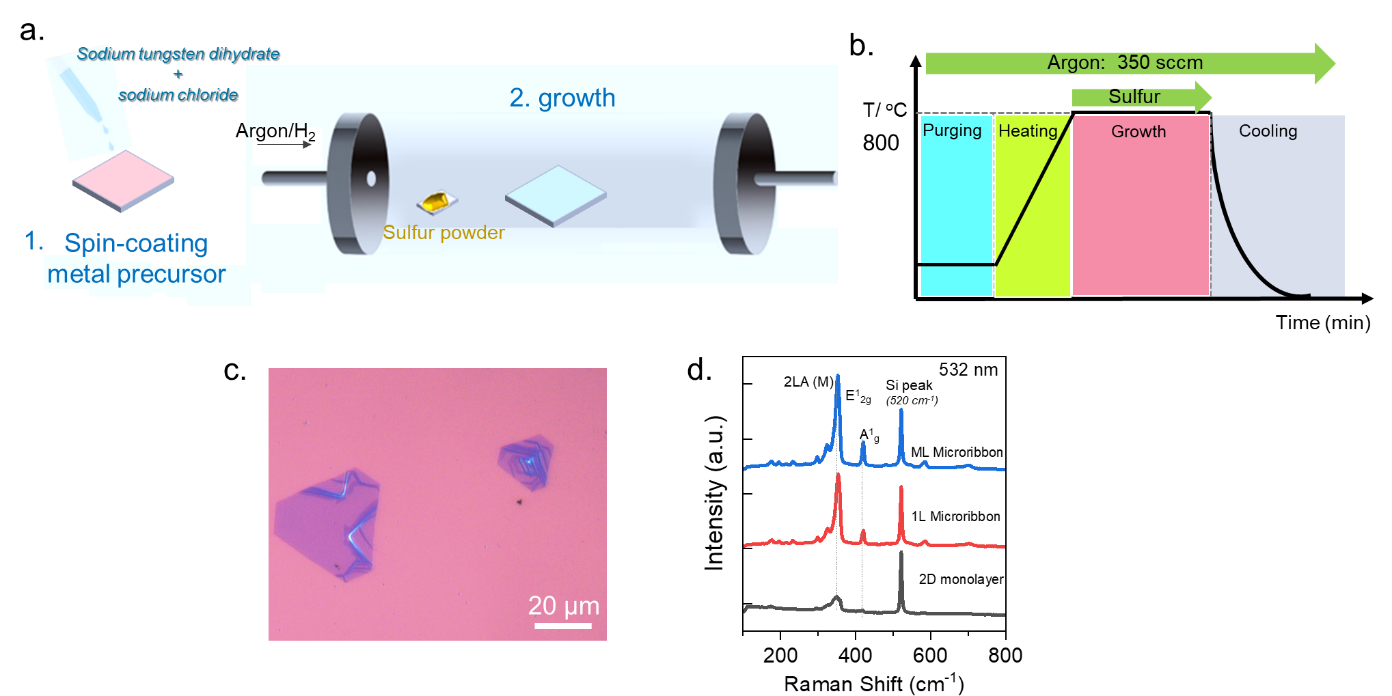


**Figure S1.** (a) Schematic illustration of the molten salt-assisted CVD process. A liquid precursor solution comprising sodium tungstate dihydrate (Na_2_WO_4_·2H_2_O) and sodium chloride is spin-coated onto a SiO_2_/Si substrate. The substrate is then placed in a tube furnace under forming gas (Ar/H_2_) with a flow of vaporized sulfur, facilitating the growth of WS_2_ microribbons on an underlying monolayer. (b) Temperature profile employed during the growth process, showing the stages: Ar purging, heating ramp, WS_2_ growth at 800 °C, and post-growth cooling. (c) Optical microscopy image of the as-grown WS_2_ monolayer and microribbon domains. Scale bar: 20 µm. (d) Corresponding Raman spectra (the out-of-plane A^1^_g_ mode, the second-order longitudinal acoustic phonon at the M-point [2LA(M)], which overlaps with the in-plane E^1^_2g_ mode and Si (substrate) peak at 520 cm^−1^) collected from the various 2D monolayer, single, and multilayer microribbons regions, confirming the 2H-phase WS_2_ peaks.

**Pipette-pulling parameters**

Pipettes were fabricated from borosilicate glass capillaries with a 1.2 mm outer diameter, 0.69 mm inner diameter, and a 7.5 cm length (Sutter Instrument, BF120-69-7.5). Single-barrel nanopipettes with nanometer-scale tip openings (~ 50 – 100 nm) were pulled using a laser pulling quartz glass capillaries using a CO_2_-laser-based puller (P-2000; Sutter Instruments) as shown in Figure S2. Single-line pulling programs were used to pipette sizes of ~54 nm and ~118 nm, respectively, with the following parameters:

| 50 nm | HEAT 780 | FIL 4 | VEL 45 | DEL 130 | PUL 110 |
| --- | --- | --- | --- | --- | --- |

| 100 nm | HEAT 780 | FIL 4 | VEL 45 | DEL 130 | PUL 100 |
| --- | --- | --- | --- | --- | --- |

**
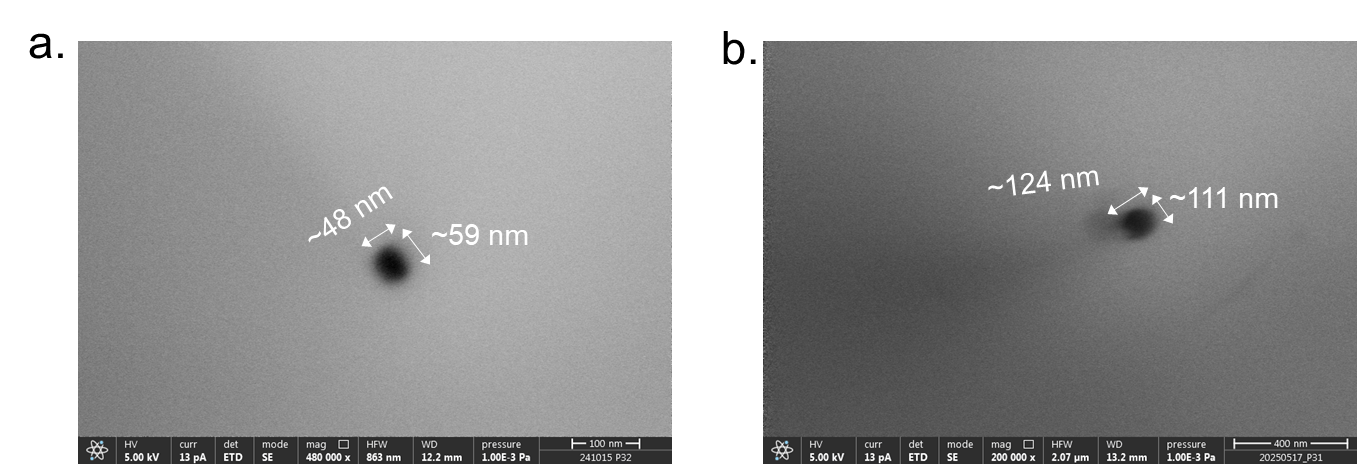
**

**Figure S2.** SEM images of nanopipettes used for the HER measurements. (a) The pipette used in Figure 2 with an average diameter of 54 ± 5 nm. (b) The pipette used in Figure 4 with an average diameter of 118 ± 7 nm.


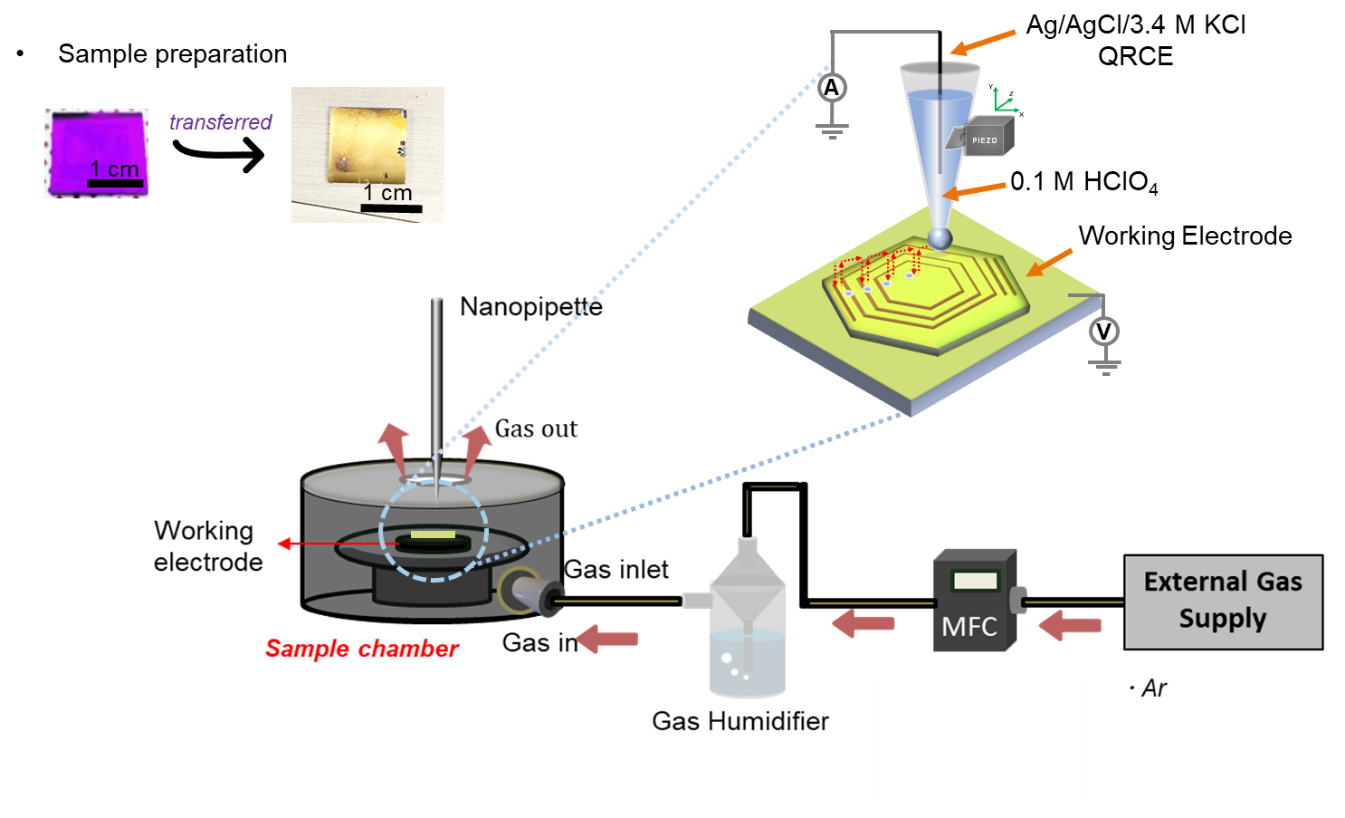


**Figure S3.** (a) Schematic illustration of the SECCM experimental setup. The system consists of a WS_2_ sample transferred onto an Au-coated SiO_2_/Si substrate serving as the working electrode. A quasi-reference counter electrode (QRCE) positioned within the nanopipette functions as both the reference and counter electrode. The sample chamber is purged with gas through a dedicated inlet, while the outlet, also serving as the nanopipette entry point, is located on the chamber lid. This configuration allows precise electrochemical measurements to be performed directly on the WS_2_ surface under controlled environmental conditions.

**Selection of electrochemically distinct regions from SECCM Maps**

The spatial regions analysed in Figures 2d and 2e were selected directly from the SECCM current map recorded at –0.4 V vs RHE. Visual inspection of the current map revealed three distinguishable regions based on spatial morphology and current response:

- Edges
- 2D monolayer regions
- Microribbons

These regions were identified based on clear differences in current density and morphological features observed directly in the electrochemical map. Edges typically showed enhanced activity and distinct boundaries (depicted in yellow), while 2D monolayers exhibited a spatially uniform higher current response (depicted in blue). Microribbons appeared as linear structures with lower current values than their surrounding 2D monolayer areas (depicted in grey color).

The selection and classification were implemented in MATLAB using a pixel-wise manual grouping approach. Each pixel was assigned to one of the three region types, enabling the extraction of localized current values for quantitative comparison. The grouped data were used to generate the averaged LSV plots (Figure 2d) and current density histograms (Figure 2e).

**
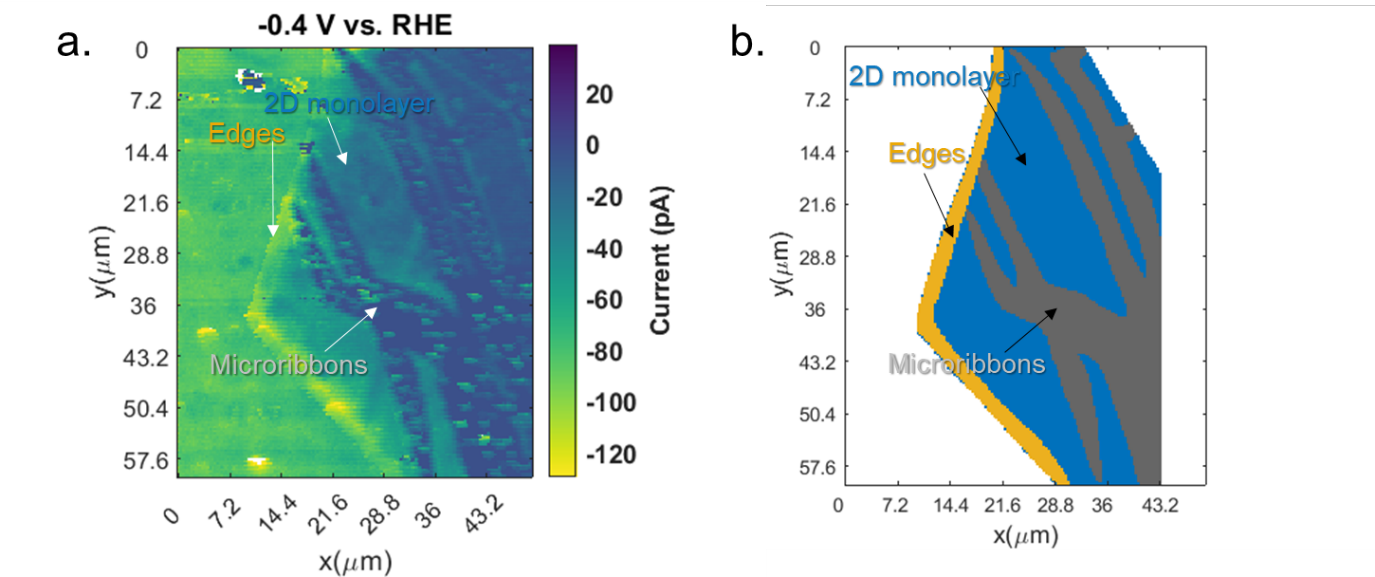
**

**Figure S4.** (a) SECCM current map recorded at –0.4 V vs RHE. (b) Classification of three representative regions selected for statistical analysis: edges (yellow), 2D monolayer (blue), and microribbons (grey). These classifications were used for extracting the averaged LSV curves and current density histograms shown in Figures 2d and 2e of the main text. Pipette diameter of ~54 nm.

**
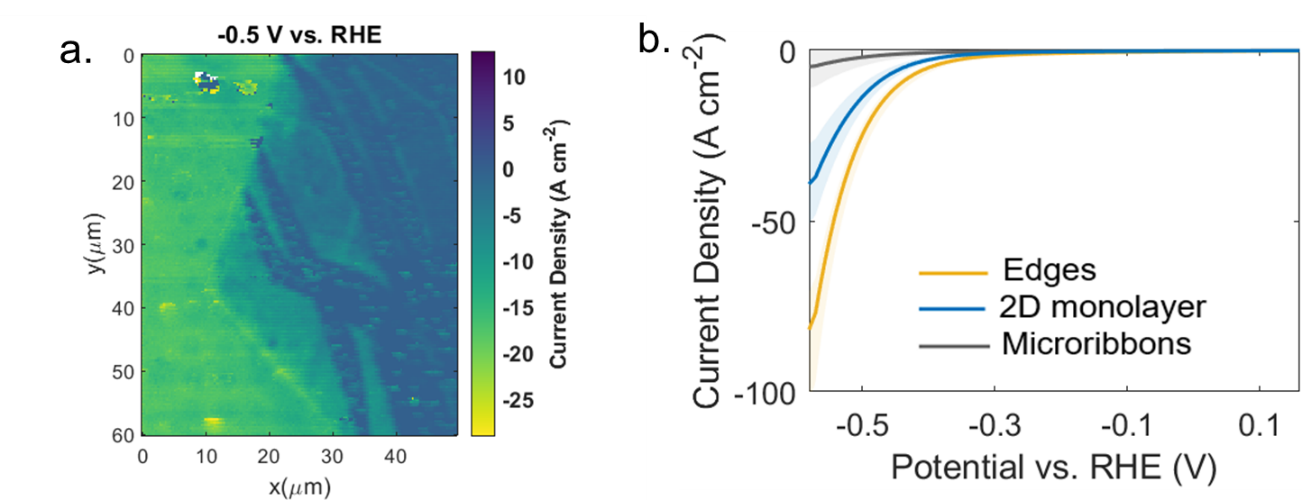
**

**Figure S5.** (a) SECCM current map at –0.5 V vs. RHE and (b) Averaged LSV curves, both normalized using the average pipette diameter of ~54 nm.

**Structural classification of WS_2_ heterogeneities via Raman intensity mapping**

The maximum Raman intensity values (~353 cm^−1^) of each location inside the flake were processed using a Gaussian mixture model (GMM) unsupervised machine learning algorithm as implemented in MATLAB, for classifying the data into three different groups. The pre-selection of the data inside the flake was done according to Figure S6a. The used algorithm captures properly the expected groups, the 2D monolayer, which shows the higher quenching of the peak is shown in blue, the 1L Microribbon, which shows moderated quenching is shown in red, and the ML Microribbon in grey.


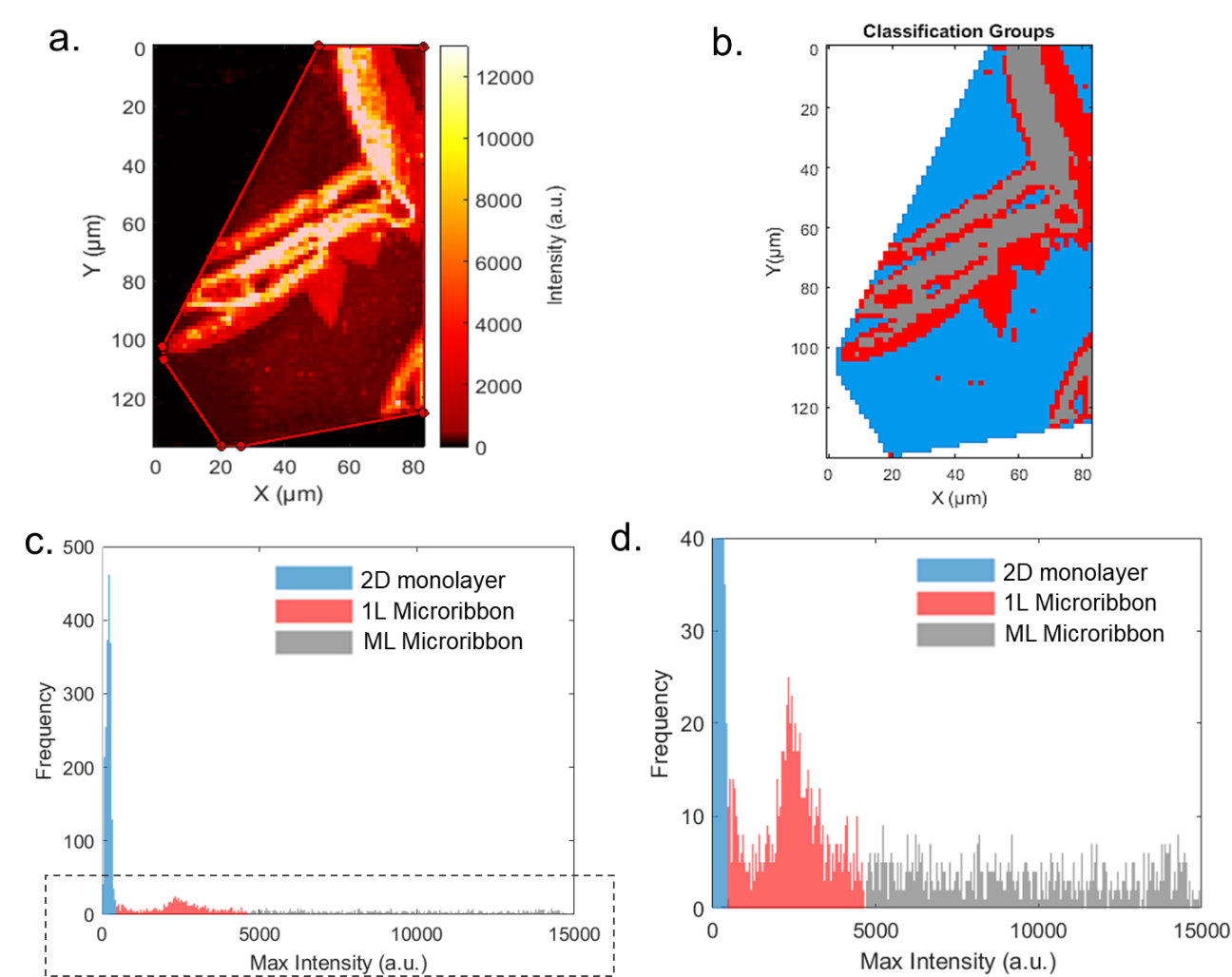


**Figure S6**. (a) Raman intensity map extracted at 353 cm^−1^, corresponding to the overlapping E^1^_2g_/2LA(M) mode of WS_2_. No Raman signal is observed on the surrounding Au substrate, confirming that the signal originates exclusively from the flake region. (b) Structural classification map delineating three distinct domains within the flake: monolayer basal plane, single-layer (1L) microribbons, and multilayer (ML) microribbons. (c) Histogram showing the distribution of maximum Raman intensities across the classified regions. (d) Zoomed-in image from (c) from the frequency range of 0 to 40, showing a clear variation of intensity across the regions. The intensity trend follows the order: ML microribbons > 1L microribbons > monolayer basal plane, consistent with stacking-induced enhancement in Raman scattering.

**Correlative alignment of Raman, SECCM maps for structural–electrochemical analysis**

The Raman and SECCM maps were performed in similar regions, but are not exactly delimited in the same region. So the Raman and SECCM data were cropped to cover a similar region of ~82 × 110 µm as pointed out by the yellow rectangle in Figure S7a and b. Raman map was plotted from 0 to 82.5 µm in the *x-axis*, and from 3.2 µm to 113.6 µm in the *y-axis*, whereas SECCM map was plotted from 30 to 112 µm in the *x-axis* and from 0 to 110 µm in the *y-axis*. The classification groups for the SECCM Map data were calculated using 2D interpolation of the Raman classification data in the selected region of interest (See Figure S7c). For the interpolation, the *x, y* grid of the selected region was translated to start at 0,0 as the origin. The grid in the Raman map is spaced by 1.5 µm in the *x*-axis and 1.6 µm in the *y-axis*, just as the original pixel size data. Whereas the grid in SECCM is spaced by 2 µm in both axes (original hopping distance). The interpolation from the grid in Raman to the grid in SECCM was performed using the simple nearest method, where for each *x, y* point in the SECCM grid, the group value was assigned from the nearest point in the Raman grid.


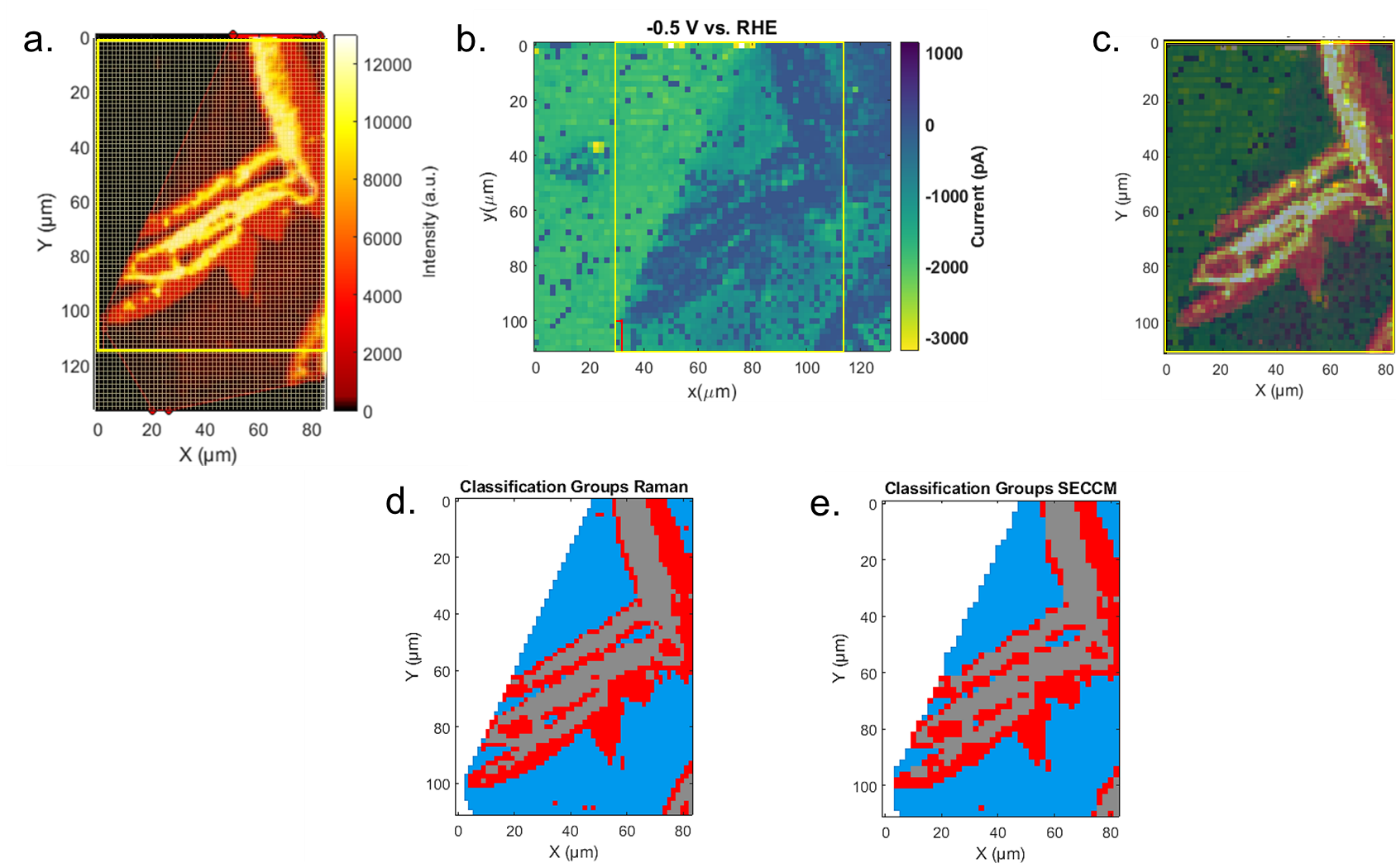


**Figure S7**. (a) Selection of the cropped region from the Raman map with grids of 1.5 µm by 1.6 µm for direct correlation with the electrochemical current map marked with a yellow box, ensuring identical spatial dimensions. (b) Cropped electrochemical current map aligned to match the selected Raman region. (c) Overlay of the Raman and SECCM maps to achieve precise spatial registration for correlative analysis. (d) Classified structural regions based on Raman data, with a spatial grid resolution of 1.5 µm along the x-axis and 1.6 µm along the y-axis. (e) Classified electrochemical activity regions based on SECCM data, with a uniform grid spacing of 2 µm along both axes.

**
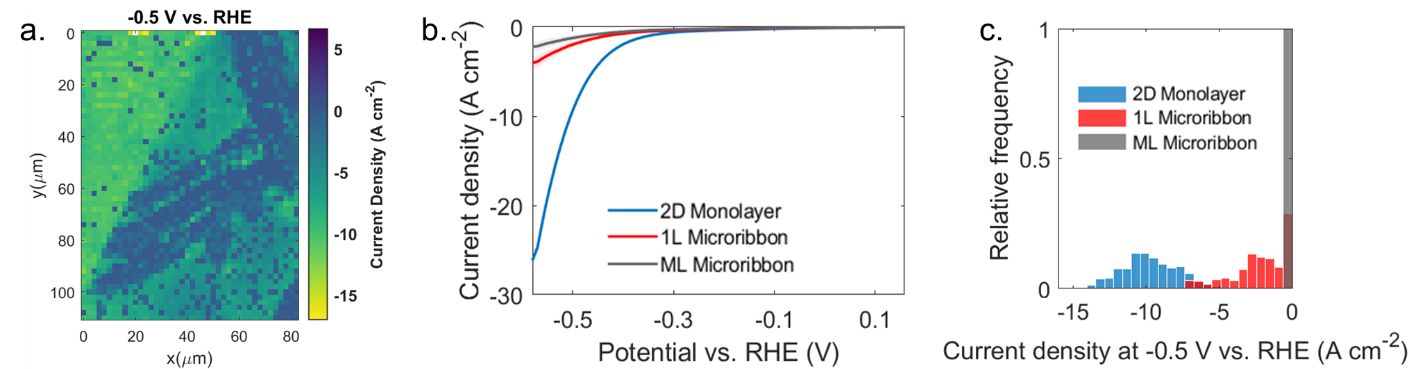
**

**Figure S8.** (a) SECCM current map at –0.5 V vs. RHE (b) Averaged LSV curves (c) Histogram of current distributions for all pixels in each region obtained by normalizing using the average pipette diameter of ~118 nm.


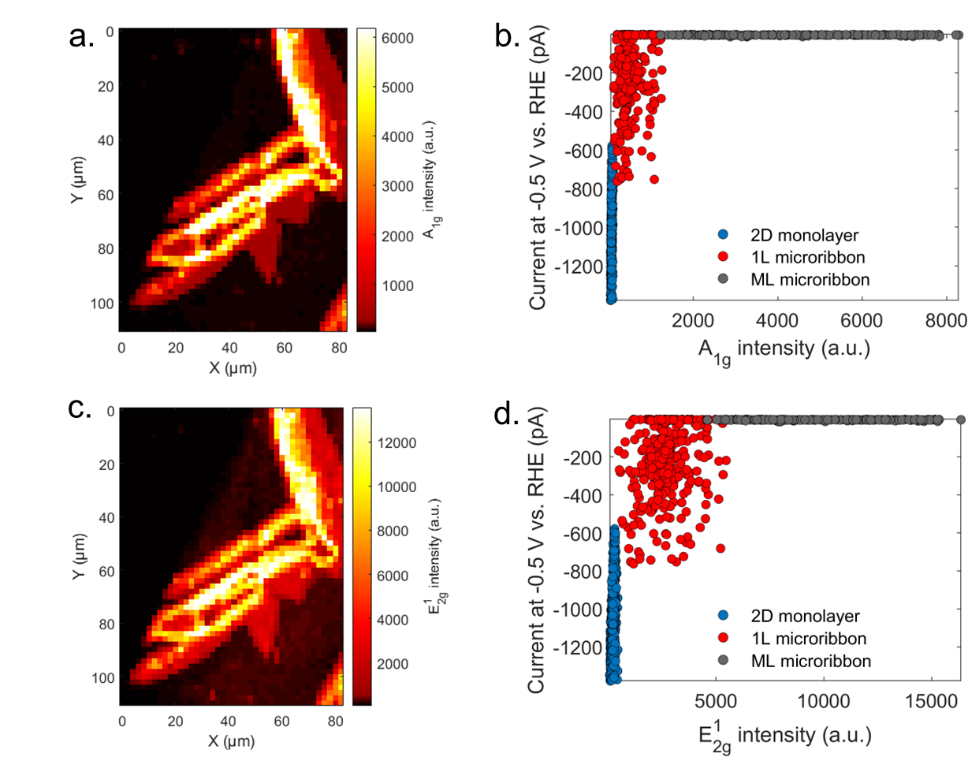


**Figure S9.** (a) A_1g_ Raman peak intensity mapping of the 2D monolayer, 1L, and ML microribbon regions. (b) Correlation between current at –0.5 V vs. RHE and A_1g_ peak intensity. (c) E^1^_2g_ Raman peak intensity mapping of the 2D monolayer, 1L, and ML microribbon regions. (d) Correlation between current at –0.5 V vs. RHE and E^1^_2g_ peak intensity.


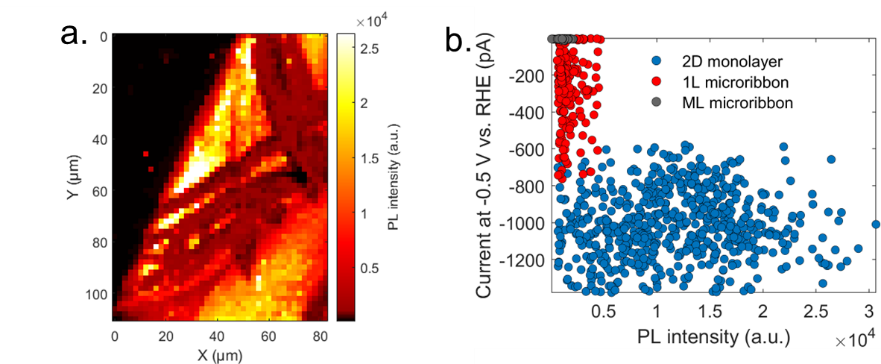


**Figure S10.** (a) PL intensity mapping of the 2D monolayer, 1L, and ML microribbon regions. (b) Scatter plot correlating current at −0.5 V vs RHE with PL intensity across the three thickness-defined regions*.*

The spatial regions analyzed in Figure 5 and Figure S11 were selected directly from the PL peak position and intensity maps. First, the 2D monolayer regions at the bottom (G1) and top (G2) of the map were chosen, as the bottom region exhibited a shift to a lower wavelength compared to the top. Within G1, two representative domains were identified using the PL intensity map, which revealed several distinct areas: one with the lowest PL intensity (A) and another with higher PL intensity (C). Similarly, two regions were selected within G2, corresponding to lower PL intensity (B) and higher PL intensity (D). The selection and classification were carried out in MATLAB using a pixel-wise manual grouping approach. This assignment is illustrated in Figure 5e and Figure S11a. The defined groupings were then used to generate the grouped scatter plots shown in Figure 5 and Figure S11.


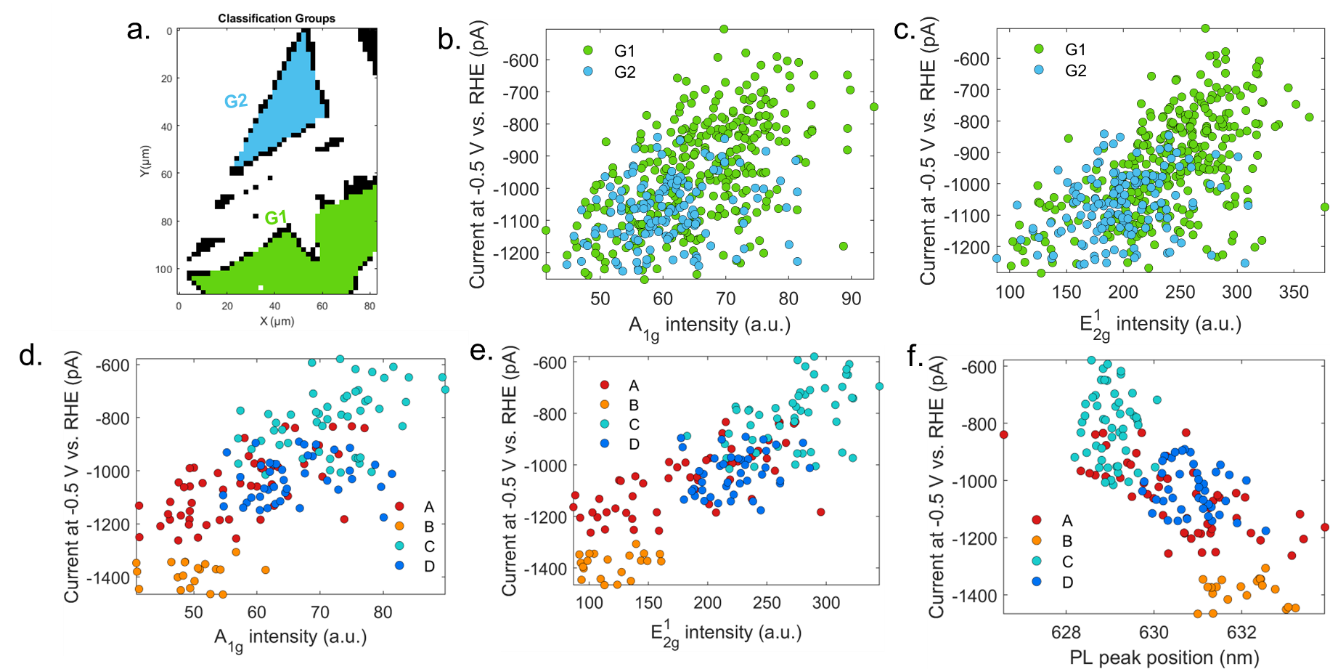


**Figure S11.** (a) Measured SECCM points located on the monolayer WS_2_ that are grouped into regions, groups G1 and G2. SECCM current at –0.5 V vs. RHE plotted against (b) Raman A_1g_ intensity and (c) E^1^_2g_ intensity for both groups. Correlation of the SECCM currents recorded at –0.5 V vs. RHE for the points marked with different colors in Figure 5d, with (d) Raman A_1g_ intensity, (e) Raman E^1^_2g_ intensity, and (f) maximum of the PL peak position.


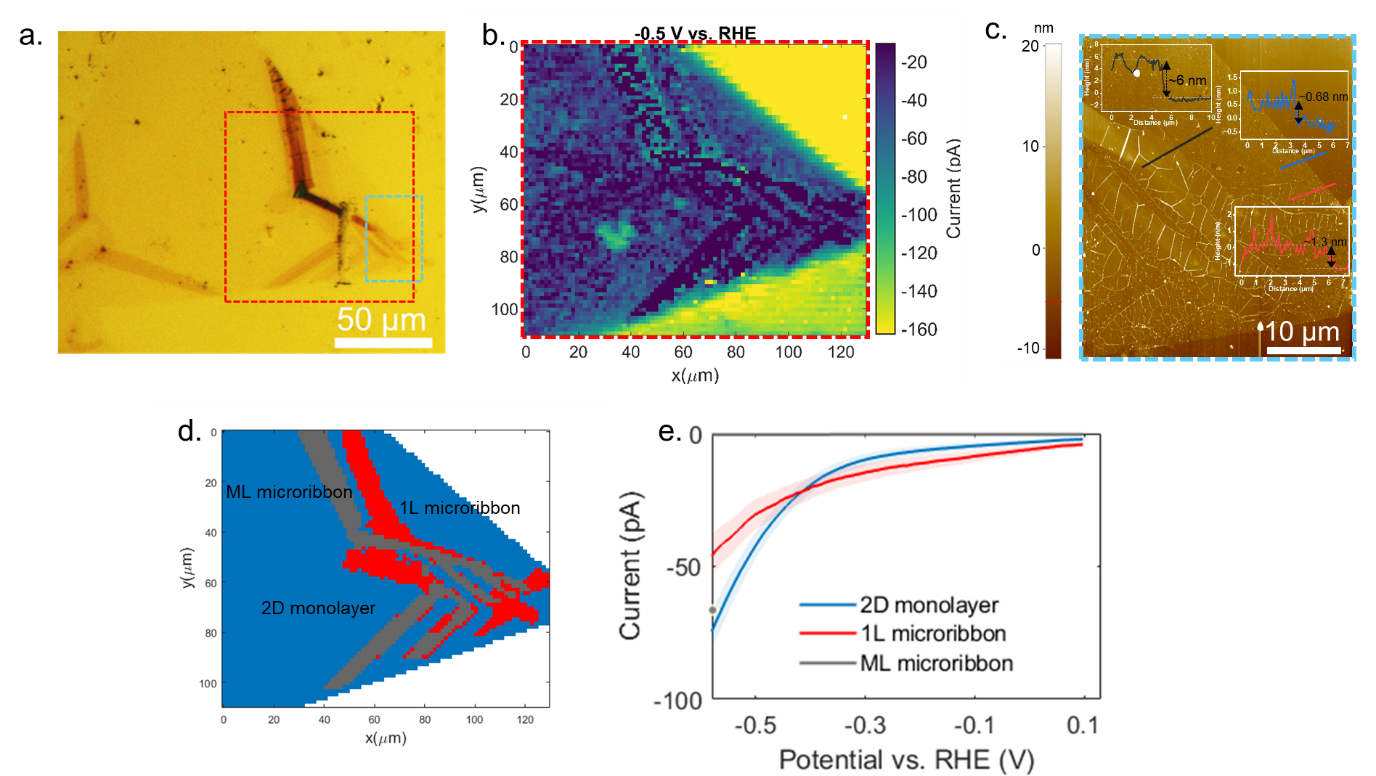


**Figure S12.** (a) Optical image of the region selected for SECCM measurements (outlined with a red dashed box). (b) SECCM activity map recorded at –0.5 V vs. RHE for WS_2_ microribbons in electrolyte (pH ~3, 0.001 M HClO_4_ + 0.1 M NaClO_4_). (c) AFM image from the light-blue boxed region in (a), showing height profiles that distinguish the 2D monolayer, 1L microribbon, and ML microribbon. (d) Structural classification map showing three distinct domains within the flake. (e) The average LSV curves for the marked region. The average pipette diameter used for the measurements was ~118 nm.
